# Genetic manipulation of Patescibacteria provides mechanistic insights into microbial dark matter and the epibiotic lifestyle

**DOI:** 10.1016/j.cell.2023.08.017

**Published:** 2023-10-26

**Authors:** Yaxi Wang, Larry A. Gallagher, Pia A. Andrade, Andi Liu, Ian R. Humphreys, Serdar Turkarslan, Kevin J. Cutler, Mario L. Arrieta-Ortiz, Yaqiao Li, Matthew C. Radey, Jeffrey S. McLean, Qian Cong, David Baker, Nitin S. Baliga, S. Brook Peterson, Joseph D. Mougous

**Affiliations:** 1 Department of Microbiology, University of Washington, Seattle, WA 98109, USA; 2 Department of Biochemistry, University of Washington, Seattle, WA 98195, USA; 3 Institute for Protein Design, University of Washington, Seattle, WA 98195, USA; 4 Institute for Systems Biology, Seattle, WA 98109, USA; 5 Department of Physics, University of Washington, Seattle, WA 98195, USA; 6 Department of Periodontics, University of Washington, Seattle, WA 98195, USA; 7 Eugene McDermott Center for Human Growth and Development, University of Texas Southwestern Medical Center, Dallas, TX 75390, USA; 8 Department of Biophysics, University of Texas Southwestern Medical Center, Dallas, TX 75390, USA; 9 Harold C. Simmons Comprehensive Cancer Center, University of Texas Southwestern Medical Center, Dallas, TX 75390, USA; 10 Howard Hughes Medical Institute, University of Washington, Seattle, WA 98109, USA; 11 Microbial Interactions and Microbiome Center, University of Washington, Seattle, WA 98195, USA

## Abstract

Patescibacteria, also known as the candidate phyla radiation (CPR), are a diverse group of bacteria that constitute a disproportionately large fraction of microbial dark matter. Its few cultivated members, belonging mostly to Saccharibacteria, grow as epibionts on host Actinobacteria. Due to a lack of suitable tools, the genetic basis of this lifestyle and other unique features of Patescibacteira remain unexplored. Here, we show that Saccharibacteria exhibit natural competence, and we exploit this property for their genetic manipulation. Imaging of fluorescent protein-labeled Saccharibacteria provides high spatiotemporal resolution of phenomena accompanying epibiotic growth, and a transposon-insertion sequencing (Tn-seq) genome-wide screen reveals the contribution of enigmatic Saccharibacterial genes to growth on their hosts. Finally, we leverage metagenomic data to provide cutting-edge protein structure-based bioinformatic resources that support the strain *Southlakia epibionticum* and its corresponding host, *Actinomyces israelii*, as a model system for unlocking the molecular underpinnings of the epibiotic lifestyle.

## Highlights

• Genetic manipulation is achieved for epibiotic Patescibacteria
• Fluorescence microscopy reveals distinct cell types in the epibiotic lifecycle
• Tn-seq uncovers the genetic basis for epibiont-host interactions
• Epibiotic growth requires a specialized secretion system and multiple adhesins

## Graphical abstract

**Figure:**
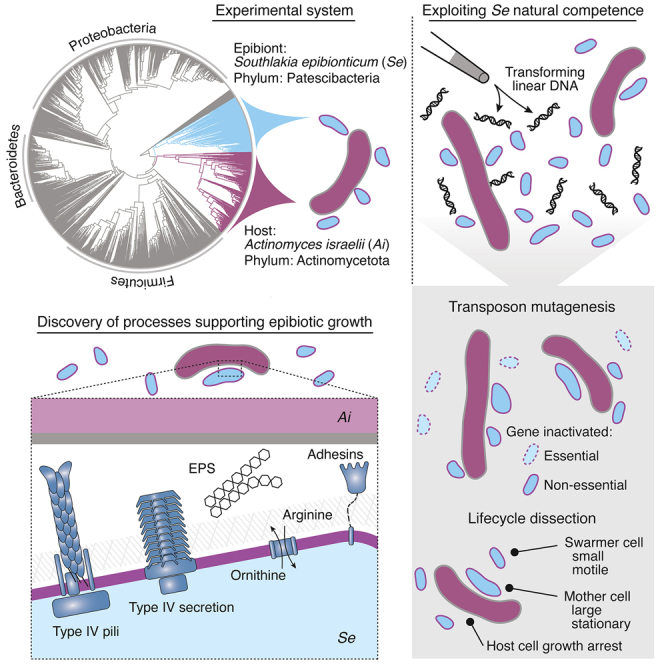

## Teaser

Discovery and exploitation of natural competence in Patescibacteria enables visualization of the bacterial epibiont lifecycle and dissection of its genetic underpinnings.

## Introduction

The vast majority of metagenomic DNA sequences obtained from microbial species-rich environmental sources are derived from Bacteria and Archaea that have not been cultivated. Conservative estimates suggest that these sequences, often referred to as microbial dark matter, represent organisms constituting approximately half of phylum level diversity within these domains.^1^^,^^2^ Microbial dark matter holds great interest as a reservoir of biosynthetic pathways and enzymes with potential for biotechnological application.^3^ In addition, understanding the functions of these genes is paramount to defining the molecular processes supporting a given ecosystem and for unraveling the physiology and cell biology of the organisms within.^4^

Patescibacteria, also known as the bacterial candidate phyla radiation (CPR), represent a large and diverse clade in the tree of bacteria with very few cultivated representatives.^1^^,^^5^^,^^6^^,^^7^ They share a number of unusual features that set them apart from most other bacteria.^8^^,^^9^ These include their small size (as little as 100–200 nm in width), reduced genomes (typically <1 Mb), and limited metabolic capability.^10^ This has led to the hypothesis that these organisms broadly share a requirement for host organisms to support their growth. Indeed, experiments show that most cultivated Patescibacteria attach to and proliferate on the surface of other bacteria—living as obligate epibionts.^11^^,^^12^^,^^13^^,^^14^^,^^15^ Genomic analyses have yielded speculation regarding the molecular functions that support the epibiotic lifestyles of Patescibacteria.^9^^,^^16^ However, owing to the phylogenetic distance separating Patescibacteria and well-characterized organisms, the function of much of their proteome cannot be predicted and a lack of genetic tools for these organisms has heretofore precluded the experimental investigation of genotype-phenotype relationships.

Among the Patescibacteria, members of the group Saccharibacteria, originally named TM7, were the first to be cultivated in the laboratory.^13^ Saccharibacteria are found in a multitude of terrestrial and marine environments; however, early interest in them stemmed from their widespread occurrence in human oral microbiomes.^17^^,^^18^^,^^19^^,^^20^ Archaeological findings show that this association dates to before the mesolithic period, and recent work links Saccharibacteria to human oral health.^17^^,^^21^^,^^22^ The growth of Saccharibacteria relies on the co-cultivation of host bacteria belonging to the class Actinomycetia within the phylum Actinomycetota, for which they exhibit strain-level specificity.^23^^,^^24^^,^^25^ Employing panels of Actinomycetia strains for Saccharibacterial enrichment has facilitated the isolation and sequencing dozens of strains.^24^^,^^26^^,^^27^ Despite this progress, the phylum remains poorly sampled, with many divergent clades uncultivated, and the extent of genetic diversity unresolved.

The genome sequences of Saccharibacteria reveal common features that provide insight into molecular functions underlying their cellular physiology and lifestyle.^2^^,^^4^^,^^13^^,^^16^^,^^28^^,^^29^ Akin to other Patescibacteria, Saccharibacteria generally lack a respiratory chain and pathways for the *de novo* generation of amino acids, nucleotides, and fatty acids.^10^ On the contrary, Saccharibacteria universally possess a relative wealth of specialized secretory mechanisms including the type II and IV secretion systems (T2SS and T4SS).^16^ These diverge significantly from related systems in bacterial pathogens that deliver toxins and effectors to eukaryotic host cells; however, it has been speculated that they function in an analogous fashion to support bacterial host co-option by Saccharibacteria.^16^ Saccharibacteria also possess type IV pili (T4P), which were implicated in twitching motility and host adhesion through the use of a small molecule inhibitor of pilus extrusion.^25^ Although such genomic analyses and experiments provide fertile ground for the formulation of hypotheses, progress toward a mechanistic understanding of the unique biology of Saccharibacteria and Patescibacteria as a whole has been stymied by a lack of genetic tools.^30^ Here, we discover that natural competence can be harnessed for genetic manipulation of Saccharibacteria. With this capability in hand, we go on to use fluorescent protein expression to conduct time-lapse microscopic analysis of the Saccharibacterial lifecycle, and we perform transposon mutagenesis to identify genes important for epibiotic growth. Our findings offer an initial mechanistic glimpse into the cellular functions encoded in microbial dark matter.

## Results

### Isolation and characterization of Saccharibacteria strains

**Figure 1:**
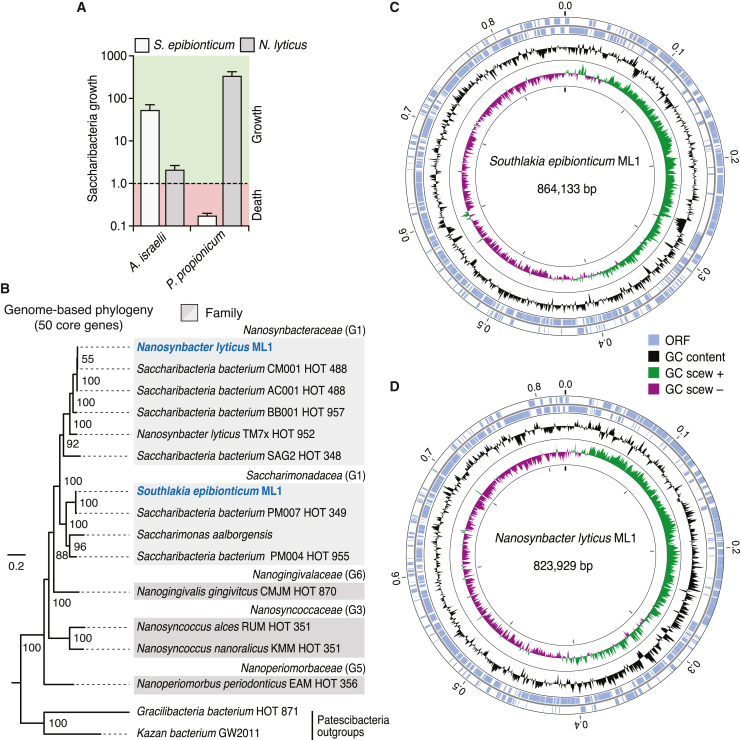
Phylogenetic placement and genome sequencing of newly isolated Saccharibacteria strains *S. epibionticum* ML1 (*Se*) and *N. lyticus* ML1 (*Nl*) (A) Maximum growth (fold change) achieved by *Se* and *Nl* during co-culture with compatible host species *A. israelii* and *Propionibacterium propionicum*, respectively, and population change (growth or death) detected at equivalent time points with an incompatible host. (B) Phylogeny constructed using 50 core, universal proteins (6,042 total amino acid positions) indicating placement of *Se* and *Nl* (blue text) within Saccharibacteria. Family names (as designated by the Genome Taxonomy Database) and groups previously designated by McLean et al. (G1, etc.) are indicated for each clade.^16^ HOT, human oral taxon. (C and D) Overview of the genome sequences of *S. epibionticum* ML1 and *N. lyticus* ML1. Data in (A) represent mean ± SD. See also Data S1–S3.

We collected, pooled, homogenized, and filtered saliva and dental plaque from volunteers and enriched this material for Saccharibacteria using the method developed by Bor et al. (detailed in STAR Methods).^26^ This led to the isolation of two strains, which exhibit distinct host specificity (Figure 1A). Phylogenetic analysis using concatenated alignments of 50 core, conserved protein sequences obtained through complete genome sequencing placed the two strains within human oral subclades of the G1 clade of Saccharibacteria (Figures 1B–1D).^31^ Based on these assignments, we named our strains *Candidatus Nanosynbacter lyticus* ML1 (*Nl*) and *Ca. Southlakia epibionticum* ML1 (*Se*). The genome sequences of the two strains additionally indicated they bear features typical of Saccharibacteria, such as genes associated with specialized secretion systems, cell surface appendages, and competence, coupled with a lack of genes required for a multitude of biosynthetic pathways and an overall reduced genome size (Figures 1C and 1D).

### Genetic manipulation of Saccharibacteria via natural transformation

We sought to develop methods for reverse genetic analyses within Saccharibacteria. Genes encoding the core components of the *com* DNA uptake system are conserved across CPR phyla, including the Saccharibacteria.^9^ These consist of ComEC, the central membrane DNA conduit, DprA, a catalyst of Rec-mediated recombination, and ComFC, which binds single-stranded DNA (ssDNA) and links import to recombination.^32^^,^^33^^,^^34^ Com proteins function in concert with T4P, which are also widely distributed in Saccharibacteria, and CPR bacteria more generally.^9^^,^^33^ Given the lack of nucleotide biosynthetic capability in CPR bacteria, it has been proposed that these systems facilitate nucleotide acquisition.^9^ Although the presence of the Com system is largely not predictive of DNA transformation in a laboratory setting,^35^ we sought to test whether exogenous DNA could be exploited for genetically manipulating Saccharibacteria.

**Figure S1:**
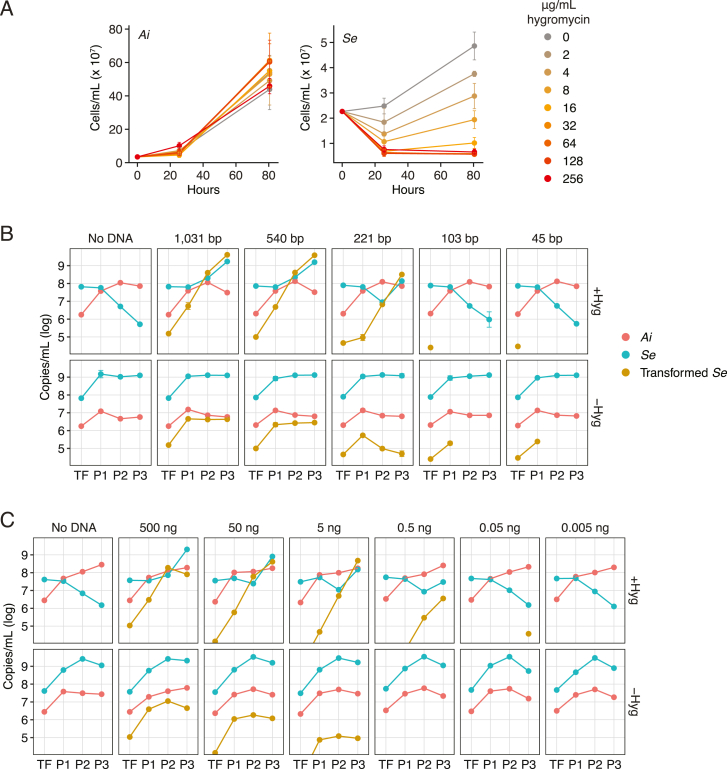
Development and optimization of the *Se* transformation protocol, related to Figure 2 (A) Growth of *Ai* (left) and *Se* (right) in co-cultures containing the indicated concentrations of hygromycin. Data represent mean ± SD. (B and C) Quantification of *Se*, *Ai*, and transformed *Se* populations over the course of our transformation protocol (see Figure 2B) with varying lengths (B) or concentrations (C) of transforming DNA. Transformations were performed in the presence (top panels) and absence (bottom panels) of selection for transformants with hygromycin.

**Figure 2:**
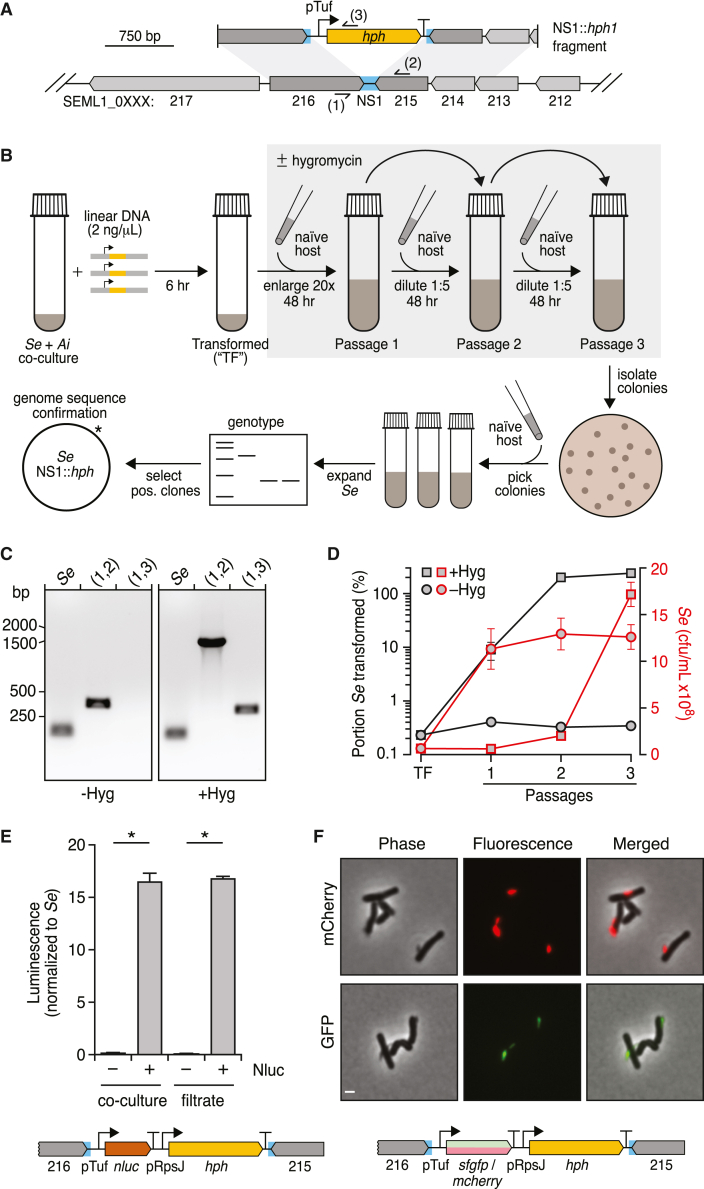
Harnessing natural transformation to generate mutant Saccharibacteria (A) Schematic depicting the intergenic neutral site (blue, NS1) targeted for insertion of a hygomycin resistance cassette (yellow) in the *Se* genome and the linear DNA fragment employed in transformation experiments. Primer binding sites used for genotyping are indicated (sites 1–3). (B) Overview of the *Se* transformation protocol. After incubation with linear DNA, *Se* + *Ai* co-cultures are enlarged concomitant with hygromycin addition and serially passage with addition of naive host at each dilution to promote *Se* growth (gray box). Clonal transformed *Se* populations were obtained by plating to isolate single colonies of *Ai* with accompanying *Se* cells, followed by growth in liquid culture, with additional *Ai*, to promote *Se* population expansion. The asterisk indicates the single insertion detected by genome sequencing. (C) PCR-based genotyping of *Se* clones obtained following transformation according to the protocol shown in (B) in the presence (right) or absence (left) of selection with hygromycin during the expansion and passaging steps. Binding sites for primers targeting NS1 (1, 2) and *hph* (3) are shown in (A). Positive control primers (*Se*) target a locus distant from NS1. (D) *Se* growth (red) and percent of *Se* transformed (black) over the course of transformation protocol depicted in (B), in the presence (squares) or absence (circles) of selection with hygromycin. (E) Luminescence production from *Se*-*Ai* co-cultures (left) or co-culture filtrates (right) in which *Se* contains a nanoluciferase expression cassette inserted at NS1 (shown at bottom). (F) Fluorescence and phase contrast micrographs of *Se*-*Ai* co-cultures in which *Se* carries an *mCherry* (top) or *sfgfp* (bottom) expression cassette inserted at NS1. Scale bar, 1 μm. Data in (D) and (E) represent mean ± SD. Asterisks indicate statistically significant differences (unpaired two-tailed Student’s t test; ^∗^p < 0.05). See also Figures S1 and S2.

As a first step toward assessing the feasibility of genetics in Saccharibacteria, we searched for antibiotics with convenient resistance determinants that potently inhibit the growth of *Se* without impacting that of its preferred host, *Actinomyces israelii* F0345 (*Ai*). These experiments revealed that across a wide range of concentrations, the aminoglycoside hygromycin fulfills these criteria (Figure S1A). Next, we designed and generated a linear cassette containing the hygromycin resistance gene (*hph*) codon optimized for *Se* flanked by the promoter and terminator regions of the *N. lyticus* TM7x *tuf* gene (elongation factor Tu), an open reading frame (ORF) predicted to be highly expressed (Figure 2A). For the insertion of this cassette, we selected an intergenic region located between two convergently transcribed ORFs, SEML1_0215 and SEML1_0216, hereafter referred to as neutral site 1 (NS1). To promote homologous recombination, approximately 1,000 bp on either side of the insertion site were added to the 5′ and 3′ ends of our cassette.

To transform *Se*, we incubated *Se*-*Ai* co-cultures with 2.0 ng/μL of our linear cassette for 6 h before initiating selection with hygromycin (Figure 2B). Naive host was added concomitantly to permit the outgrowth of successfully transformed *Se* cells. Reasoning that transformation may be inefficient, we passaged these co-cultures twice, at 48-h intervals, with continued hygromycin selection and the addition of naive host. Cultures were then diluted and plated to obtain colonies, which were selected and propagated with naive host without selection before genotyping (Figure 2B). The latter step was included to bottleneck the *Se* population and facilitate the isolation of clonal populations. Remarkably, each *Se*-infected culture we tested—accounting for the majority of colonies selected—contained our synthetic cassette inserted at the expected location (Figure 2C). Whole genome sequencing confirmed these PCR results, and it further showed that cassette integration occurred without introducing off-target mutations.

**Figure S2:**
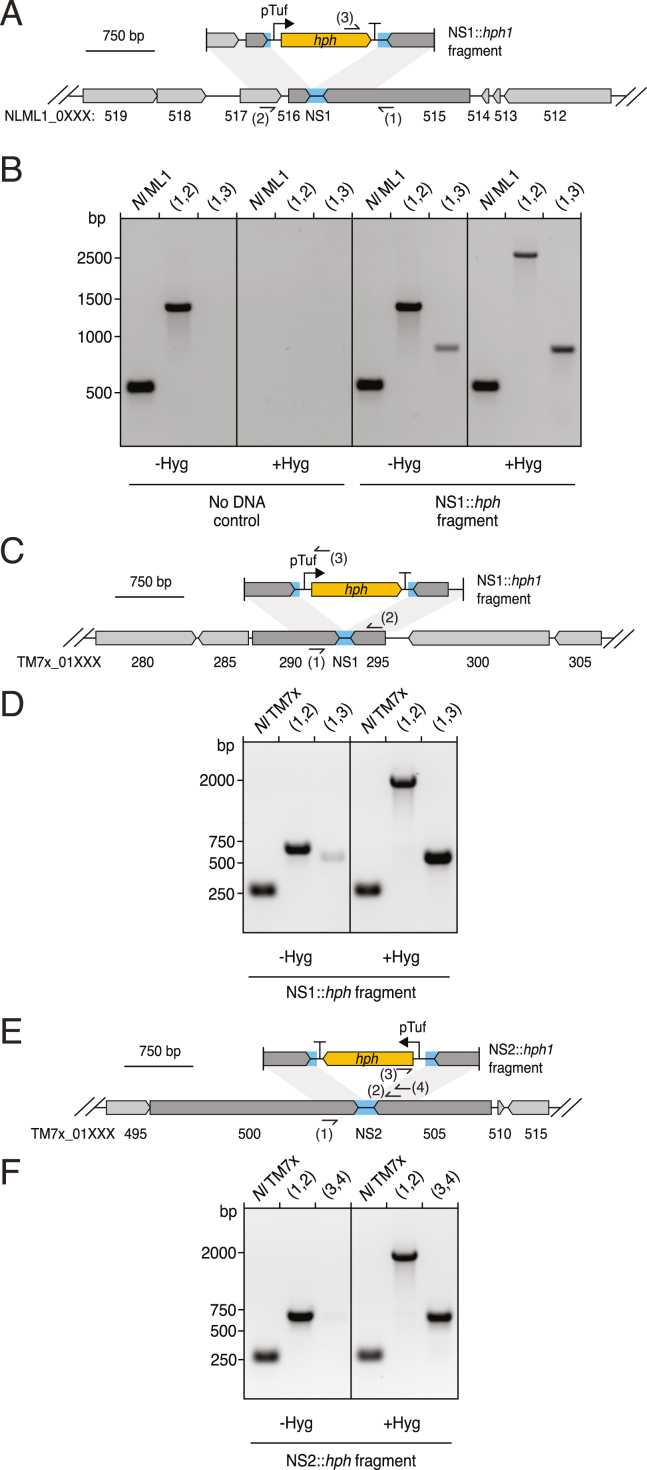
Transformation of two strains of *Nanosynbacter lyticus*, related to Figure 2 (A, C, and E) Schematics depicting the intergenic neutral sites (blue, NS1 and NS2) targeted for insertion of a hygomycin resistance cassette (yellow) in the *N. lyticus* ML1 (*Nl* ML1) (A) or *N. lyticus* TM7x (*Nl* TM7x) (C and E) genomes and the linear DNA fragment employed in transformation experiments with these strains. Primer binding sites used for genotyping are indicated (sites 1–3). (B) Genotyping of *Nl* ML1*-P*. *propionicum* co-cultures transformed with the linear DNA fragment depicted in (A) (right) or parallel negative control co-cultures with no DNA added (left) at the end of passage 4 (see STAR Methods) with primers targeting NS1 (1,2) or *hph* (3). Positive control *Nl* ML1 primers target a genomic locus distant from NS1. (D and F) Genotyping of *Nl* TM7x-*Schaalia odontolytica* co-cultures transformed with the linear DNA fragment depicting in (C) or (E), respectively, at the end of passage 4. Cultures were grown in the presence (right) or absence (left) of continuous selection with hygromycin. Primers targeted NS1 (D) or NS2 (F) (1,2) or *hph* (3). Positive control *Nl* TM7x primers target a genomic locus distant from NS1 and NS2.

Quantitative PCR (qPCR) analysis of total *Se*, transformed *Se*, and *Ai* at regular intervals during our transformation procedure demonstrated that approximately 0.2% of *Se* contains the integrated cassette by the conclusion of the initial incubation period (Figure 2D). Although *Se* levels remain low through the second passage under selection with hygromycin, all surviving *Se* cells bear the cassette at this time point. In the final passage, the population of *Se* continues to maintain the cassette and expands markedly, far surpassing levels of the host (Figure S1B). In the absence of hygromycin, similar quantities of initially transformed *Se* are observed; however, this small proportion fails to expand despite overall robust growth of *Se*. We observed similar transformation behavior using lengths of DNA with homology to the insertion site flanking regions as short as 221 bp (Figure S1B) and with as little as 0.02 ng/μL (Figure S1C). Finally, to probe the generality of our methods, we identified predicted NSs within *Nl* and the previously published Saccharibacterial strain *N. lyticus* TM7x.^13^^,^^36^ We then subjected these strains to an analogous transformation protocol. Genotyping of transformed populations by both PCR and whole genome sequencing of *N. lyticus* TM7x purified clonal populations indicated cassette insertion at the desired locations also occurred within these strains (Figure S2).

The ability to introduce heterologous DNA into *Se* has numerous foreseeable applications, one of which is the expression of reporter genes that allows *Se* to be distinguished and studied within the context of co-culture with their hosts. To explore this possibility, we designed and generated NS1 insertion cassettes containing genes encoding nanoluciferase, mCherry, and green fluorescent protein (GFP) under the control of the *tuf* promoter and upstream of *hph* driven by a second predicted strong promoter of *N. lyticus* TM7x, that of *rpsJ* (Figures 2E and 2F). Using our transformation protocol, we obtained clonal populations of hygromycin-resistant *Se* containing each of these genes. Luminescence assays and fluorescence microscopy demonstrated robust activity of each reporter gene (Figures 2E and 2F). We did not detect their activity in host cells, indicating the feasibility of achieving specific manipulation of *Se* in the context of a co-culture.

**Figure 3:**
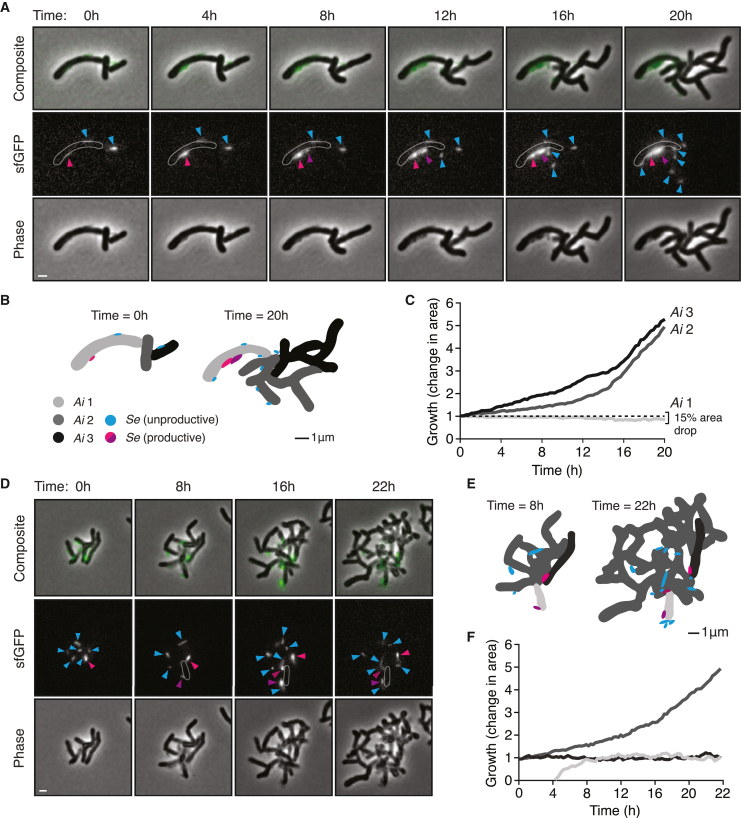
Fluorescent protein expression and quantitative microscopy enable tracking of the *S. epibionticum* lifecycle (A and D) Snapshots captured at the indicated time points from time-lapse fluorescence and phase contrast microscopy of GFP-expressing *Se* grown in co-culture with *Ai*. Arrows indicate example *Se* cells exhibiting productive (pink, purple) and non-productive (blue) interactions with *Ai* cells. White outlines in the fluorescent channel depict an *Ai* cell affected by *Se* infection (*Ai* 1). Scale bar, 1 μm. (B and E) Omnipose-generated segmentation of *Se* and *Ai* cells depicted in (A) and (D), at the start (left) and end (right) of the 20- or 22-h growth period. (C and F) Growth of individual *Ai* cells as impacted by productive (light gray) or non-productive *Se* cells (black, dark gray). Colors correspond to cell masks shown in (B) and (E). Time-lapse fluorescence microscopy videos from which (A) and (D) derive, as well as additional raw and annotated videos of *Se*-*Ai* co-cultures, are open for access via https://mougouslab.org/data.

The expression of fluorescent proteins within *Se* provided the opportunity to visualize CPR bacterial growth with extended, time-lapse fluorescence imaging. Over the course of 20 h, both *Se* and *Ai* populations—deposited from co-cultures at a low *Se*:*Ai* ratio onto an agar substrate containing growth media—showed clear evidence of expansion (representative time-lapse fluorescence microscopy videos are open for access via https://mougouslab.org/data). Apparent T4P-mediated motility of *Se* was also observed, as reported by Xie et al.^25^ Our time-lapse imaging further captured features of the Patescibacteria lifecycle at unprecedented spatiotemporal resolution. For instance, we could distinguish productive (*Se* growth supporting) versus non-productive (*Se* adhered without concomitant growth) interactions and directly measure their respective impact on individual host cells (Figure 3). Additionally, we observed productively adhered mother cells producing small swarmer cell progeny via repeated polar budding and the differentiation of a subset of these progeny into mother cells. Altogether, these findings show that natural transformation can be exploited to genetically manipulate Patescibacteria in a directed manner and open a window into the distinctive biology of this largely unexplored group of organisms.

### Tn-seq identifies genes essential for epibiotic growth

**Figure S3:**
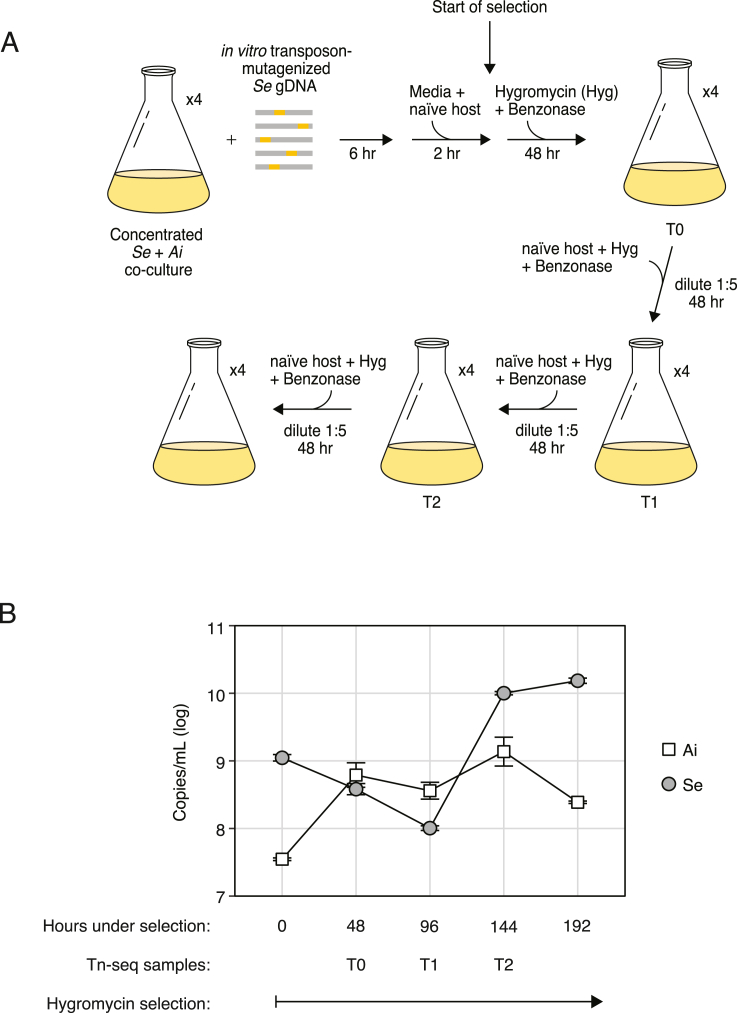
Population dynamics of *Se* and *Ai* during transposon mutagenesis, related to Figure 4 (A) Schematic depicting the protocol employed for transposon mutagenesis of *Se*. (B) Quantitative PCR-based measurements of *Se* and *Ai* levels during the course of the transposon mutagenesis experiment. Data in (B) represent mean ± SD.

The ability to genetically manipulate Saccharibacteria enables myriad avenues of investigation. As a first step toward genetic dissection of the *Se* epibiotic relationship with *Ai*, we conducted transposon-insertion sequencing (Tn-seq) within *Se* during growth on *Ai*. To this end, we performed *in vitro* Tn5-based transposition on purified *Se* genomic DNA, repaired gaps as described by Manoil and colleagues, and used sequencing to confirm high-frequency, homogeneous insertion across the genome.^37^ This DNA was then used to transform *Se*, with slight modifications from our basic transformation protocol (see STAR Methods). Most notably, we elected to increase the scale of the experiment to account for our previously measured transformation efficiency and thereby avoid population bottlenecking following the onset of selection (Figure S3A). We collected three samples for Tn-seq analysis, an initial sample following the transformation and recovery period (T0), and two additional samples representing the population after 48 h serial passages with the addition of naive host (T1 and T2). Measurements of *Se* and *Ai* levels at each of these time points revealed a drop in *Se* levels prior to T1 that is not observed at similar time points in transformations targeting NS1, suggesting that a majority of *Se* cells received lethal mutations (Figure S3B). Our measurements also showed that, as expected, *Ai* levels drop relative to those of *Se* at later passages, such that by T2, *Se* outnumbers *Ai* by approximately 10-fold.

**Figure 4:**
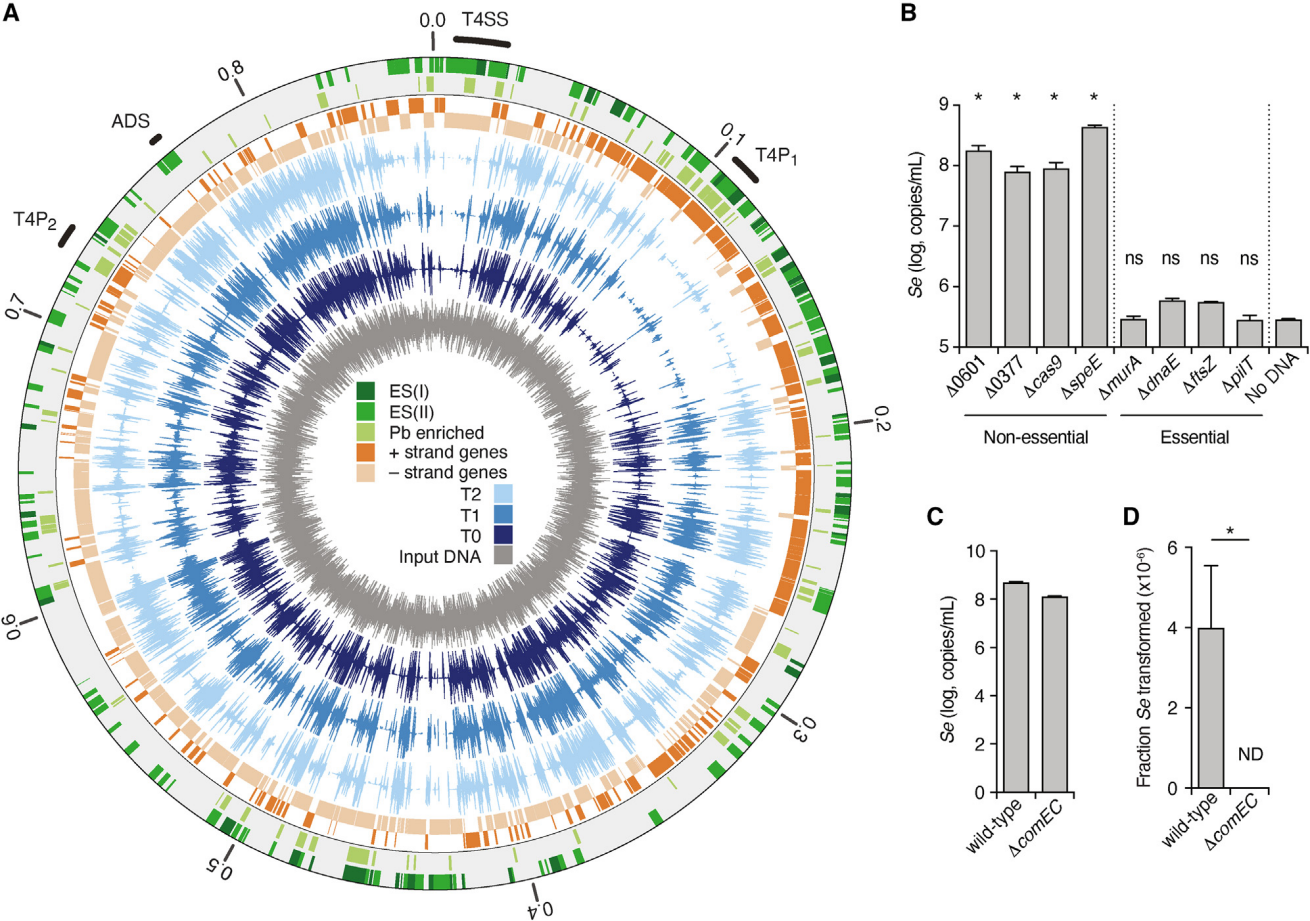
Identification of genes important for fitness of *S. epibionticum* during co-culture with *Ai* identified by Tn-seq (A) Overview of normalized transposon-insertion frequency across the *Se* genome detected in input DNA used for mutagenesis (dark gray), and from samples collected 48 h after the onset of selection (T0, dark blue) and subsequent outgrowth time points (T1-T2, shades of blue). Genes encoding proteins belonging to Patescibacteria-enriched protein families (Pb enriched) and class I and II essential genes (ES I and II) are indicated in the outer circles (shades of green). The locations of the essential arginine deiminase system (ADS) genes, T4SS genes, and two loci containing T4P genes (T4P_1_ and T4P_2_) are indicated outside of the circle. (B) *Se* population levels detected in *Se*-*Ai* co-cultures following transformation with constructs designed to replace the indicated genes with *hph*. (C and D) Total *Se* population (C) and proportion transformed (D) following transformation with an unmarked cassette targeted to NS1 in the indicated strains of *Se*. See also Figures S4 and S5 and Table S1. Data in (B)–(D) represent mean ± SD. Asterisks indicate statistically significant differences (B, one-way ANOVA followed by Dunnett’s compared to no DNA control; D, unpaired two-tailed Student’s t test; ^∗^p < 0.05, ns, not significant). See also Figures S3 and S4 and Table S1.

Insertion site sequencing of our Tn-seq samples indicated a high density of insertions across the genome at each of the time points collected (40,382 unique sites among T0-T2; Figure 4A). These data were then applied to the Hiden Markov Model (HMM)-based essential gene identification algorithm within the TRANSIT software package.^38^ This method compares the depth of sequencing reads obtained at each insertion site and calls essential genes based on the deviation of this value across a given ORF from the genome average. In total, we identified 295 essential genes in *Se*, which fell into two classes (1) genes called as essential at T0 (61 genes, class I) and (2) genes called essential only after T0 (234 genes, class II) (Figure 4A; Table S1). KEGG analysis showed that class I genes are enriched in genetic information processing (53%). Many class II genes also belong to this category (34%); however, a higher proportion of genes in this group fall within metabolism and signaling categories. It is worth noting that one-third and one-half of class I and II genes, respectively, could not be classified by this method.

To validate our Tn-seq results, we selected two class I and two class II essential genes, along with four non-essential control genes, and designed constructs to replace each with an *hph* expression cassette via a double-crossover recombination event. The constructs shared equivalent length flanking sequences to enable the direct comparison of their behavior in our transformation protocol. We assessed the fitness impact of inactivating each gene by quantifying *Se* populations following transformation and outgrowth under selection with hygromycin. For the control genes not predicted to contribute to fitness, we achieved robust levels of *Se* growth by this time point, comparable with that achieved when introducing the *hph* expression cassette at NS1 (Figure 4B). In contrast, in transformations targeting each of the four predicted essential genes examined, *Se* failed to proliferate, consistent with the inactivation of these genes strongly impacting fitness. Together, these findings provide confirmation that our Tn-seq analysis successfully identified the relative fitness contributions of *Se* genes during co-culture with *Ai*.

We noted that genes encoding homologs of *com* system components ComEC, ComF, and DprA were not among those genes defined as contributing significantly to *Se* fitness in our Tn-seq study. This finding suggests that this DNA competence machinery is not required for acquiring nucleotides to support *Se* growth, as had been previously suggested.^9^ To determine whether the *com* system of *Se* instead functions to mediate natural transformation, we generated a *Se* strain in which the *comEC* ORF is replaced by the *hph* expression cassette (*Se* Δ*comEC*::*hph*). To assess whether this mutation affects *Se* transformation, we measured the efficiency of inserting a second, unmarked cassette at NS1. At the conclusion of the transformation protocol, insertion at the NS1 site was only detectable in the wild-type background, supporting the hypothesis that the *com* system mediates natural transformation in this species (Figures 4C and 4D).

### A distinctive set of essential cellular features characterize the Patescibacteria lifestyle

**Figure S4:**
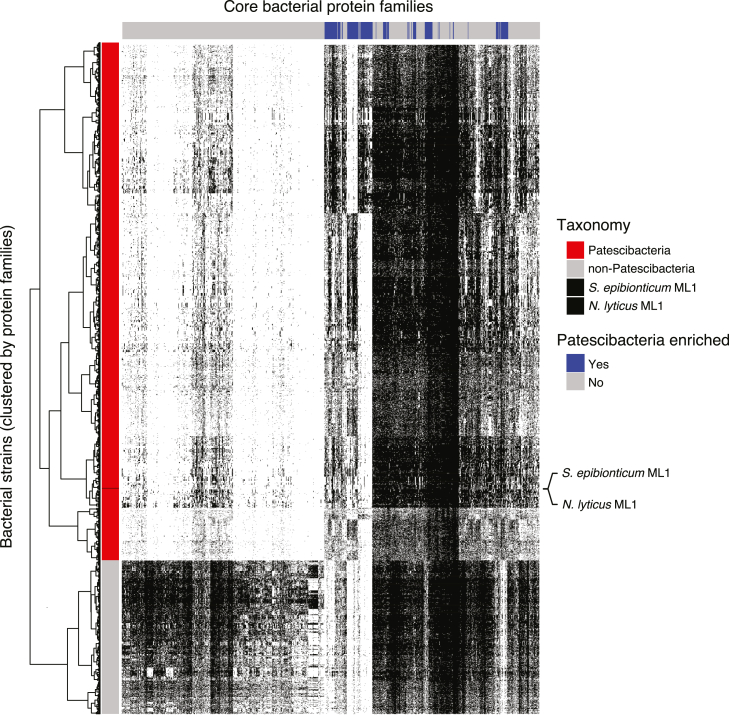
Distribution of 921 core protein families across Patescibacteria and other bacterial genomes, including *Se* and *N. lyticus* ML1, related to Figure 4 Columns represent core families (derived from Meheust et al.^9^) and rows represent individual genomes from the indicated bacterial groups. Patescibacteria-enriched protein families indicated at top (blue), and dendrogram at left represents clustering of bacterial strains based on protein family content.

The number of essential genes we identified in *Se* (295) falls within the typical range of essential genes in free-living bacteria. This is despite *Se* lacking the genes that typically comprise a significant proportion of essential gene sets, such as those participating in the biosynthesis of fatty acids, nucleotides, and amino acids. We hypothesize that *in lieu* of these biosynthetic pathways, the *Se*-essential gene set includes Patescibacteria-specific genes that specifically enable its unique host-cell-associated lifestyle. Banfield and colleagues previously leveraged the large number of Patescibacteria genomes available from metagenomic and traditional genome sequencing datasets (n = 2,321) to define Patescibacteria-enriched protein families.^9^ To gain insight into which of these are essential for *Se* growth on *Ai*, we identified *Se* proteins belonging to these families and cross-referenced these against the essential gene list obtained from our Tn-seq experiment (Figures 4A and S4). This revealed that 73 of the 116 Patescibacteria-enriched genes within *Se* are essential for growth, a dramatic enrichment (∼5-fold) relative to their proportion in the genome.

The phylogenetic distance of *Se* from well-studied bacterial systems poses a challenge for standard genome annotation methods and thus limited our capacity to leverage our Tn-seq data to define Patescibacteria-specific processes important for epibiotic growth. To improve functional predictions associated with ORFs in the *Se* genome, we applied a battery of sequence and structure-based computational tools. ProtNLM is a natural language model trained on UniProt to predict protein names given a sequence.^39^ Applying ProtNLM to the *Se* proteome yielded functional annotations for 337 of the 855 proteins (ProtNLM score ≥ 0.5, Table S2). This sequence-based annotation was further improved by mapping each protein to Pfam domains using hmmscan, detecting Pfam domains in an additional 270 proteins, 245 of which could be assigned functions (Table S2).^40^^,^^41^

**Figure 5:**
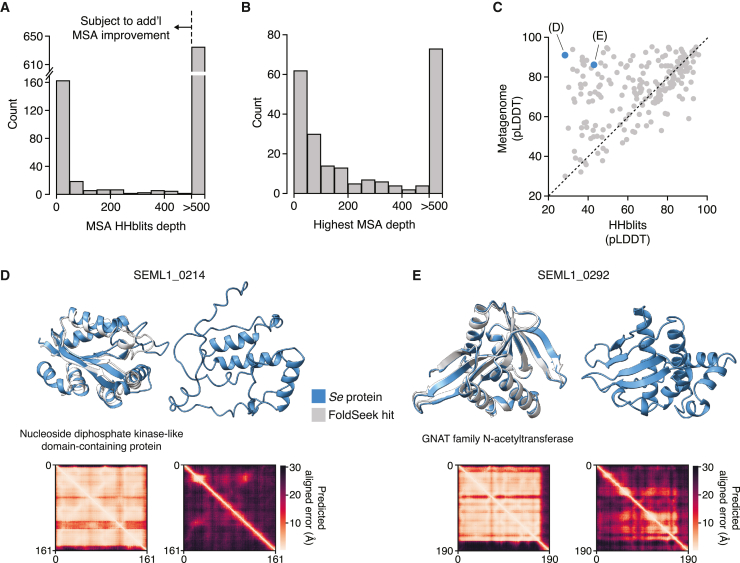
Inclusion of extensive metagenomic data in MSAs enables proteome-wide AF modeling of *S*. *epibionticum* protein structures (A) Histograms depicting MSA depths obtained for *Se* proteins using HHblits. (B) Maximum depths obtained for *Se* protein MSAs that initially contained <500 sequences. Additional sequences were sourced from metagenomic sequence databases and incorporated into MSAs using Jackhmmer or Phmmer (see STAR Methods). (C) Comparison of the AF confidence metric (pLDDT) determined using Hhblits or Jackhmmer/Phmmer (metagenome)-generated MSAs for *Se* proteins with initially shallow MSAs (<500). *Se* proteins shown in (D) and (E) are highlighted in blue. (D and E) Example *Se* protein structure models and associated predicted alignment matrices obtained using shallow (right) or metagenomic sequence-improved (left) MSAs. *Se* protein models (blue) are aligned to models from top Foldseek (FS) hits (light gray, AF database50 numbers A0A1F6S045, D and A0A7W4ES58, E), when available. The annotation in (D) and (E) derives from the best FS hit. Some structures are trimmed to highlight the alignment. See also Figures S5A–S5C and Table S2.

**Figure S5:**
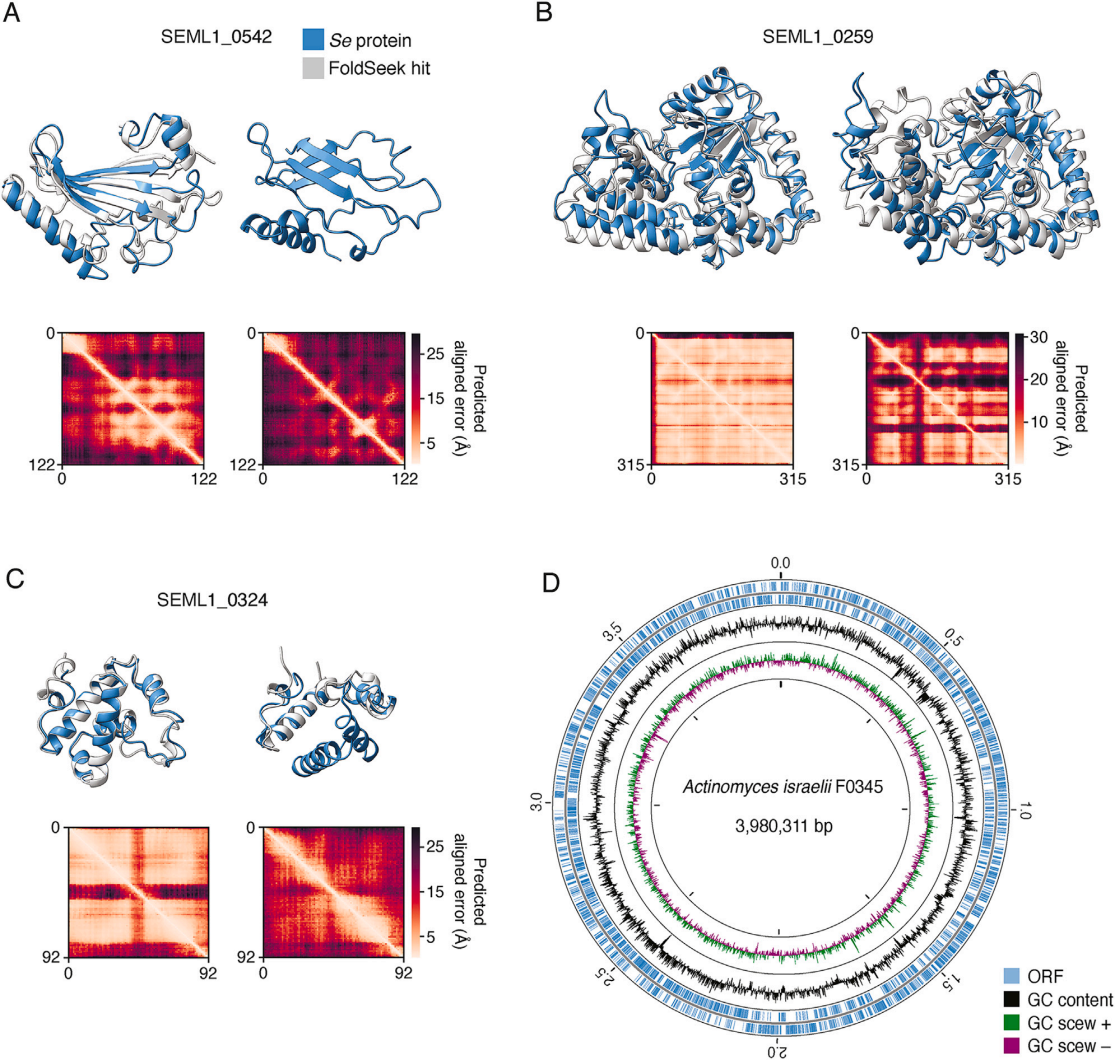
Structural models for the *Se* proteome generated using metagenomic sequence enriched MSAs and the *Ai* genome, related to Figures 5 and 6 (A–C) Example *Se* protein structure models and associated predicted alignment matrices obtained using shallow (right) or metagenomic sequence-improved (left) MSAs. *Se* proteins models (blue) are aligned to models for top FS hits (light gray, AF database50 numbers A0A8B1YQG7, A; A0A660M2Z7, metagenome and R7KEI0, shallow, B; A0A563D6X1, metagenome and A0A563CX08, shallow, C), when available. Some structures are trimmed to highlight the alignment. (D) Overview of the genome sequence of *A. israelii* F0345.

To complement our sequence-based annotations, we took advantage of recent advances in protein structure prediction to conduct genome-wide structure-based homology analyses of the *Se* proteome.^42^^,^^43^ Generation of structural models using AlphaFold (AF) relies on evolutionary information extracted from multiple sequence alignments (MSAs).^43^ For nearly 25% of the *Se* proteome (220 proteins), our initial MSAs based on HHblits searches of UniRef and BFD (the default databases used by AF) were too shallow for high confidence structure prediction (<500 sequences post-filtering; Figure 5A).^44^^,^^45^^,^^46^ To improve the MSA depth for these proteins, we implemented a hidden Markov model (HMM)-based approach to identify and align additional homologs for each protein from multiple metagenomic datasets. This resulted in considerably deeper MSAs (>500 sequences) for an additional 9% of the proteome (Figure 5B). Using the highest depth MSA obtained for each protein, we obtained AF models for >99% of the predicted *Se* proteome. For comparison, we also computed AF models using the original 220 MSAs that contained <500 sequences and compared the model confidence metric obtained using these and the improved depth MSAs. Particularly, for low-confidence models obtained using MSAs generated with the default databases (average pLDDT < 50), we found that the use of the deeper MSAs for structural prediction resulted in substantial model confidence improvement (Figure 5C). The structure model improvements enabled by using extensive metagenomic databases for MSA generation were further underscored by structural homology search results obtained using Foldseek (FS).^47^ For some proteins, the improved structural models led to the identification of structural homologs for proteins that initially had no FS matches passing our cutoffs, whereas for others, the changes in the overall predicted structure led to the identification of different and more closely aligning top matches (Figures 5D, 5E, and S5A–S5C). In total, using FS, we were able to identify similar structures for 89% of modeled proteins (761/852, Table S2).

The incorporation of structural information resulted in functional predictions for 70 *Se* proteins that had none assigned by sequence-based approaches (Table S2). However, many of the AF models with similar structures identified for *Se* proteins are uncharacterized (12%), providing little information regarding function. Moreover, unrelated proteins may share similar structures due to convergence, and additional evidence is usually needed to assess the homologous relationships. Domain Parser for AF Models (DPAM) is a recently developed tool that parses structural domains from AF-modeled structures and integrates both structure and sequence searches to map protein domains to evolutionary classification of protein domains (ECOD).^48^^,^^49^ Using DPAM, we detected homologous ECOD domains for 80% of the predicted *Se* proteins (Table S2). This included 31 proteins for which no function was assigned by other methods. In supplementary material accompanying this report, we provide alignments for each mapped *Se* protein domain, along with links to corresponding Protein Data Bank identifiers (PDB IDs) and ECOD classifications (https://conglab.swmed.edu/ECOD_Se/Se_ECOD.html).

**Figure 6:**
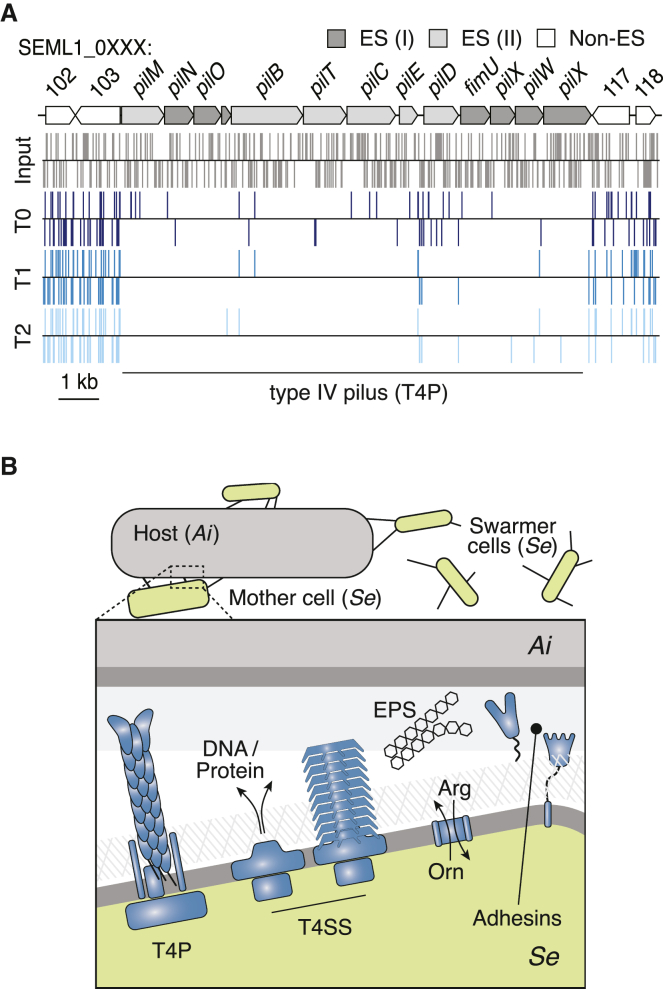
Unusual essential genes of *S*. *epibionticum* encode numerous envelope-associated functions predicted to mediate host-cell interaction (A) Schematic of genes encoding the core T4P components (T4P_1_ locus, top), and locations of transposon insertions detected in input mutagenized DNA (gray) and Tn-seq samples T0-T2 (shades of blue). Gene essentiality is indicated by shading (class I essential genes [ES I], dark gray; class II essential genes [ES II], light gray). (B) Model of the *Se*/*Ai* interface during a productive infection, depicting selected envelope-associated functions found to be essential in our Tn-seq screen. These include macromolecular structures predicted to mediate adhesion (T4P) and DNA or protein delivery (T4SS), protein adhesins and the ADS system for arginine catabolism. Mother and swarmer cell designations derive from the analysis shown in Figure 3. See also Figure S5D. EPS, extracellular polysaccharide; Orn, ornithine; Arg, arginine.

Aided by our improved annotation of the *Se* genome, we identified cellular functions that are uniquely essential to the epibiotic lifestyle (Figures 6A and 6B). Consistent with its adhesion to and reliance on a host cell for sustenance, many of these are localized outside of the *Se* cytoplasm. This includes T4P, which typically mediate surface attachment and twitching motility. A prior study utilizing quercetin, a small molecule inhibitor of pilus retraction, reported that T4P are important for host attachment in the Saccharibacterial species *Leucosynbacter cicadicola*.^25^ We find that the majority of genes associated with T4P function (SEML1_0104-0116, SEML1_0689-0693, and SEML1_0758-0768) are class I essential genes, indicating that disrupting this appendage is immediately detrimental to *Se* survival and further suggesting that T4P remain critical even after a productive interaction is established (Figures 4 and 6A; Table S1). Of note, in naturally competent bacteria, T4P work in concert with the *com* system to bind and mediate the uptake of extracellular DNA.^50^^,^^51^ Our observation that *Se* cells lacking *comEC* are viable suggests that the essentiality of T4P is unrelated to its role in competence.

A second essential cell envelope-associated machinery we identified is an apparent T4SS (SEML1_0006-0019; Figure 6B). This specialized secretion system can participate in myriad functions but is most often implicated in the direct transfer of DNA (conjugation) or effector proteins to neighboring prokaryotic or eukaryotic cells. Our structure-based annotation shows that the subunit composition of the *Se* T4SS is unusual, but most closely resembles that found in conjugative T4SSs of Gram-positive bacteria (Table S2). A notable distinction is the presence of a series of predicted pilin subunits in *Se*, which are essential and encoded within the T4SS gene cluster; Gram-positive conjugative T4SSs generally lack a pilus. Other differences include the lack of recognizable coupling and relaxase proteins in the *Se* T4SS. These proteins directly participate in and are required for DNA transport, suggesting that the T4SS of *Se* may not function in this manner and may instead export proteins or serve in an alternative capacity.

In addition to macromolecular machines, we found an assortment of other essential cell envelope-associated proteins whose predicted function is not typically associated with critical cellular processes in bacteria (Figure 6B). Several of these proteins contain domains implicated in polysaccharide binding or degradation, including the lectin-like VCBS domain (SEML1_0891), the cellosome-associated dockerin domain (SEML1_0315), and the glycan-binding fibronectin III domain (SEML1_0890). We speculate that the proteins containing these domains target host-cell-associated glycans, either for promoting adhesion or for degradative purposes. An additional essential protein and two adjacent genes classified as conferring a growth deficit if inactivated are predicted components of extracellular polysaccharide (EPS) biosynthesis machinery (SEML1_0081-0083). Therefore, an EPS synthesized by *Se* may also contribute to productive host-cell interaction.

The arginine deiminase system (ADS) is an adenosine triphosphate (ATP)-generating catabolic pathway prevalent in mammalian-adapted Saccharibacteria species.^36^ A prior study demonstrated that arginine supplementation to *N. lyticus* TM7x-*Schaalia odontolytica* co-cultures permits acid neutralization via ammonia production, supporting the viability of both strains during acid stress.^36^ The authors of this study put forth a model in which hosts lacking ADS benefit from and are specifically permissive to colonization by Saccharibacteria containing the pathway. In *Se*, the ADS consists of ArcA, a fusion of arginine deiminase and ornithine carbamoyltransferase, ArcC, a carbamate kinase, and ArcDE, an arginine/ornithine antiporter (SEML1_0801-0803); all were classified as essential in our Tn-seq study (Table S1). Interestingly, in contrast to the proposed model for ADS function in Saccharibacterial-host cell interactions, sequencing of the *Ai* genome revealed intact homologs of each ADS gene (Figure S5D). Taken together with our Tn-seq data, this observation suggests that in some co-culture pairs, the organisms may compete for arginine. In summary, the uniqueness of the epibiotic lifestyle of *Se* is borne out by the unusual collection of essential functions revealed by our Tn-seq data.

## Discussion

Scientists have been aware of Patescibacteria in environmental samples for many years; however, our understanding of this group of organisms has lagged.^11^ One major challenge is their apparent strict requirement for host bacteria, the identity of which cannot currently be determined *a priori*, thus adding significant complexity to Patescibacteria isolation.^12^^,^^26^^,^^52^^,^^53^ It is foreseeable that methods bringing to bear both experimental and computational approaches could provide predictions of host-epibiont associations in the future. Here, we addressed a second impediment to studying Patescibacteria—a lack of genetic tools for their manipulation. Indeed, despite streamlined methods for isolating Saccharibacteria on host Actinobacteria that have been employed by several laboratories, this latter challenge has, to date, prohibited a molecular dissection of their epibiotic lifestyle.^24^^,^^25^^,^^53^ Our discovery that natural transformation can facilitate targeted mutagenesis in Saccharibacteria opens the door to direct interrogation of genotype-phenotype relationships within these Patescibacteria; however, challenges remain. The most fundamental hurdle is that factors required for the Saccharibacteria-host cell interaction are also required for viability. Indeed, our Tn-seq analysis led to the identification of numerous essential genes not typically required for bacterial growth that encode predicted functions consistent with their involvement in mediating the interaction between *Se* and *Ai*. In analogous situations, researchers overcome this challenge using assorted conditional inactivation strategies (e.g., modulated expression, temperature sensitivity, and inducible degradation). Although it is conceivable that such methods could be implemented in Saccharibacteria, a powerful approach in this system may involve exploiting host genetics to identify gene interactions that suppress otherwise lethal mutations in the epibiont.

The small genome of Saccharibacteria stands in contrast to the apparent complexity of their lifecycle. Xie et al. proposed a model for the epibiotic growth of Saccharibacteria that consists of four stages: T4P-mediated infection, growth, bud formation, and asymmetric division.^25^ Our time-lapse microscopy generally supports this model; however, it further revealed facets of the Saccharibacteria-host interaction, which were not previously captured. Although the aforementioned model implies uniform progression, our data suggest the lifecycle is more complex. We find that only a small subset of *Se* achieve productive infections and that after an extended enlargement period, these cells rapidly bud a large number of highly motile swarmer cell progeny (>10 in a 20-h period observed in many instances). Quantitative analyses of our data additionally permitted us to link productive and non-productive *Se* infections with corresponding host cell outcomes. We observed a striking inverse relationship between *Se* growth and that of *Ai*; cells productively infected with as few as one *Se* not only failed to divide but diminished in size, whereas those infected unproductively by multiple *Se* readily proliferated.

Our microscopy observations suggest a potential division of labor within *Se* populations, wherein one subpopulation is devoted to reproduction, and a second, motile subpopulation searches for new compatible host cells. Operating under the assumption that the Saccharibacteria-Actinobacteria relationship is chiefly one of parasitism, which we maintain is not fully resolved in the literature, then the relevant prevailing evolutionary theory predicts that *Se* fitness is dependent on both the rate at which it causes new infections and the duration of those infections.^54^ The reproductive strategy of *Se* appears consistent with this framework; release of progeny cells destined to infect other host cells tempers host cell burden and prolongs the infection, while concurrently maximizing *Se* reproduction. A similar outcome could be achieved if *Se* employed active mechanisms to block superinfection, reminiscent of those utilized by phage; however, swarmer cell production has the added advantage of increasing *Se* dispersal in the face of low host cell densities.^55^ Interestingly, most nascent swarmer cells we observed do not themselves establish a productive infection within our 20-h observation period, despite close proximity and apparent adherence to host cells. It may be that the time required to establish a productive interaction varies, and many of these cells would go on to become productive. It is also conceivable that infected host cells defend against secondary infections, perhaps even inducing a defensive state in neighboring kin cells. Future studies coupling quantitative microscopy with genetics to further dissect the *Se*-*Ai* interaction will no doubt address these questions and shed light on this fascinating interphyla relationship.

The set of essential genes we identified in *Se* differs markedly from those described in other bacteria. One striking omission from the list is its predicted F-type ATP synthase, all the components of which are dispensable for growth on *Ai*. This raises the question of why *Se* maintains the capacity to assemble this large macromolecular machine. Like most Patescibacteria, *Se* lacks a respiratory chain and likely grows via anaerobic fermentation.^10^^,^^11^ It likely generates ATP by substrate-level phosphorylation, including via the arginine deiminase pathway, which we found is essential.^56^ During fermentative growth, ATP synthase can operate in reverse, hydrolyzing ATP and extruding protons, which is thought to contribute to maintaining the proton motive force (PMF).^57^ However, the lack of essentiality of ATP synthase in *Se* suggests that it is not the primary driver of the PMF. It has been hypothesized that the close physical proximity maintained between Saccharibacteria and their host could allow the epibionts to harness the host-generated proton gradient, either to facilitate solute transport or to fuel ATP synthesis.^10^ However, we find that growth of *Ai* is arrested rapidly upon productive association with *Se*; it is unclear how long such non-growing host cells maintain their proton gradient. Interestingly, several oral *Streptoccocus* species rely on proton extrusion by an ATP synthase acting in reverse to resist the acid stress generated by cariogenic oral bacteria.^58^ Given that Saccharibacteria are common oral cavity inhabitants, we speculate that they may employ the complex in a similar fashion.

We anticipate that the methods and genetic tools presented here will facilitate molecular-level characterization of Saccharibacteria and Patescibacteria more broadly. In this regard, one key question is the extent to which natural competence is active and able to be similarly exploited across Patescibacteria. The ability to manipulate Patescibacteria outside of Saccharibacteria, particularly those with phylogenetically distinct hosts and inhabiting diverse niches, should aid in elucidating the core requirements of the epibiotic lifestyle. Regardless of the precise methods utilized, genetic manipulation of Patescibacteria will open the door to phenotypic studies of the rich reserves of microbial dark matter these organisms contain, potentially revealing unprecedented biological mechanisms.

### Limitations of the study

Although the methods we developed for genetic manipulation of Saccharibacteria unlock many previously inaccessible routes of investigation, they are nevertheless subject to limitations. Chief among these is the reliance on a single antibiotic-resistance cassette, which precludes facile construction of strains with multiple mutations and may limit the breadth of organisms that can be targeted. Additionally, we have yet to develop an approach for generating unmarked mutants and have not established whether genetic manipulation via natural transformation can be applied to Patescibacteria outside of Saccharibacteria. Beyond the technical limitations of our approach, applying the methods we describe toward genotype-phenotype investigations of *Se* is limited by (1) the difficulty of distinguishing genes required for basic physiology from those encoding host interaction determinants and (2) screening under *in vitro* growth conditions that differ from those encountered in nature (e.g., the oral cavity for *Se*).

## STAR★Methods

### Key resources table

**Table:** 

REAGENT or RESOURCE	SOURCE	IDENTIFIER
**Bacterial and virus strains**		

*Southlakia epibionticum* ML1	This study	N/A
*Nanosynbacter lyticus* ML1	This study	N/A
*N. lyticus* TM7x	He et al.^13^	N/A
*Actinomyces israelii* F0345	Dewhirst et al.^59^	N/A
*A. odontolyticus* F0309	Batty^60^	N/A
*Schaalia odontolytica* XH001	He et al.^13^	N/A
*A. urogenitalis* S6-C4	Nikolaitchouk et al.^61^	N/A
*Actinomyces sp.* F0386	Dewhirst et al.^59^	N/A
*Propionibacterium propionicum* F0230	Dewhirst et al.^59^	N/A

**Chemicals, peptides, and recombinant proteins**		

Instagene matrix	Bio-Rad	Cat#732-6030
SsoAdvanced Universal SYBR Green Supermix	Bio-Rad	Cat#1725272
EZ-Tn5 Transposase	Biosearch Technologies	Cat#TNP92110
Benzonase Nuclease	Sigma	Cat#E1014

**Critical commercial assays**		

DNeasy Blood & Tissue Kit	Qiagen	Cat#69506
Nano-Glo Luciferase Assay System	Promega	Cat#N1110

**Deposited data**		

The complete genome sequences of *S. epibionticum* ML1, *N. lyticus* ML1 and *A. israelii* F0345	This study	GenBank: PRJNA957798
Transposon insertion sequencing data	This study	NCBI Sequence Read Archive: PRJNA957798

**Oligonucleotides**		

Primers used in this study are listed in Table S3.	N/A	N/A

**Recombinant DNA**		

DNA fragments used in this study are listed in Table S4.	N/A	N/A

**Software and algorithms**		

Geneious Prime 2023.1.2	Geneious, Software, Newark, New Jersey, USA	https://www.geneious.com; RRID:SCR_010519
Prism 9 for macOS	GraphPad, Software, La Jolla, California, USA	https://www.graphpad.com; RRID:SCR_022798
Adobe Illustrator 27.3.1	Adobe Systems Incorporated, San Jose, California, USA	https://www.adobe.com/products/illustrator; RRID:SCR_010279
UCSF ChimeraX version 1.6.1	UCSF, Software, San Francisco, California, USA	https://www.cgl.ucsf.edu/chimerax/; RRID:SCR_015872
Trycycler v0.5.2	Wick et al.^62^	N/A
Filtlong v0.2.1	https://github.com/rrwick/Filtlong	N/A
Flye v2.9	https://github.com/fenderglass/Flye/releases/tag/2.9. Accessed 4 October 2021	N/A
Raven v1.8.1	Vaser and Šikić^63^	N/A
Miniasm v0.3r179	Li^64^	N/A
Minipolish v0.1.3	https://github.com/rrwick/Minipolish/blob/main/miniasm_and_minipolish.sh	N/A
Polypolish v0.5.0	Wick et al.^62^	N/A
MaSuRCA v4.0.9	Zimin et al.^65^	N/A
PROKKA v1.14.5	Seemann^66^	N/A
BGME	Criscuolo and Gribaldo^67^	N/A
IQtree	Trifinopoulos et al.^68^	N/A
Omnipose	Cutler et al.^69^	N/A
TRANSIT suite	DeJesus et al.^38^	N/A
ProtNLM	Gane et al.^39^	N/A
hmmscan	Eddy et al.^40^	N/A
KofamKOALA version 2023-04-01	Aramaki et al.^70^	N/A
HHblits	Remmert et al.^44^	N/A
phmmer and jackhmmer	Eddy^40^	N/A
AlphaFold	Jumper et al.^43^	N/A
FoldSeek v6.0	van Kempen et al.^47^	N/A
Domain Parser for AlphaFold Models (DPAM)	Zhang et al.^49^	N/A

**Other**		

5 μm mixed cellulose esters (MCE) membrane	Millipore	SMWP04700
5 μm syringe filters, Surfactant-Free Cellulose Acetate and Cellulose Acetate	Thermo Scientific	723-2545
0.1mm zirconia/silica beads	BioSpec	Catalog#11079101z
Mini-BeadBeater-16	Biospec	Model 607
Breath-Easy sealing membrane	Sigma	Z380059

### Resource availability

#### Lead contact

Further information and requests for resources and reagents should be directed to and will be fulfilled by the lead contact, Joseph Mougous (mougous@uw.edu).

#### Materials availability

Plasmids and bacterial strains generated in this study are available upon request from the lead contact.

### Experimental model and study participant details

#### Strains, media and growth conditions

Saccharibacteria strains employed in this work include *Southlakia epibionticum* ML1 and *Nanosynbacter lyticus* ML1, both isolated in this study, and *Nanosynbacter lyticus* TM7x.^13^ Host bacterial strains used include *Actinomyces israelii* F0345, *A. odontolyticus* F0309, *Schaalia odontolytica* XH001 (XH001),^13^ *A. urogenitalis* S6-C4, *Actinomyces sp.* F0386 and *Propionibacterium propionicum* F0230 (*Pp*).^59^^,^^60^^,^^61^ *Ai* and *Pp* host bacteria mono-cultures, *Se-Ai* co-cultures and *Nl* ML1*-Pp* co-cultures were routinely grown statically under an atmosphere of ambient air supplemented with 5% CO_2_ at 37°C or anaerobically with shaking at 37°C using the GasPak EZ Anaerobe Container System with Indicator (BD 260626 and 260001) in TSY media (30 g/L tryptic soy and 5 g/L yeast extract) or TSYR media (TSY media supplemented with 10 mM arginine), or anaerobically on TSBY agar plates (30 g/L tryptic soy, 5 g/L yeast extract, 15 g/L agar, supplemented with 5% (v/v) horse blood). XH001 host bacteria mono-cultures and *Nl* TM7x-XH001 co-cultures were grown at 37°C anaerobically in Brain Heart Infusion (BHI) media or on BHI agar plates. For selection of hygromycin-resistant Saccharibacteria, hygromycin was used at 150 μg/mL. *Se-Ai* and *Nl* ML1*-Pp* co-cultures were stored at -80°C in TSY or TSYR supplemented with 10% (v/v) Dimethylsulfoxide (DMSO). *Nl* TM7x-XH001 co-cultures were stored at -80°C in BHI supplemented with 25% (v/v) glycerol.

### Method details

#### Isolation of Saccharibacteria strains *Se* ML1 and *Nl* ML1

Isolation of Saccharibacteria strains in co-culture with host bacteria was carried out as essentially as previously described.^53^^,^^71^ Anonymous volunteers aged over 18 years provided oral samples for Saccharibacteria isolation. Supragingival plaque samples were collected with toothpicks and dispersed in 1 mL of Maximum Recovery Diluent (MRD; Peptone 1.0 g/L, Sodium Chloride 8.5 g/L, pH 7.0) buffer. 5 mL of saliva was collected by voluntary expectoration into sterile 50 mL conical tubes. All saliva samples and plaque samples were then pooled into 20 mL MRD. Pooled samples were then vigorously resuspended by vortexing and filtered with a 0.22 μm filter, and the flowthrough was collected. Residual bacteria in the sample tube and filter were collected by washing once with 10 mL MRD, filtered again, and combined with the previous flowthrough. Saccharibacteria present in filtrates were pelleted by centrifuging at 60,000 rcf for 1 hr at 4°C. Supernatant was removed, and pellets were resuspended in 1 mL MRD. The presence of Saccharibacteria in these samples were confirmed by PCR, using phylum specific primers^71^ (Table S3). The collected Saccharibacteria were then added to mono-cultures of a panel of five potential host species (*A. odontolyticus*, *A. urogenitalis*, *Actinomyces sp.* F0386, *Ai*, and *Pp*) and the cultures were passaged every 24 or 48 hr. The presence of Saccharibacteria cells in the final cultures was confirmed by qPCR with universal Saccharibacteria primers (Table S3) and microscopic imaging. The cultures were then streaked on TSY or TSBY agar, isolated colonies of host bacteria were tested for Saccharibacteria by PCR, and positive colonies were re-cultured in liquid medium and stored as clonal co-cultures.

To create purified suspensions of Saccharibacteria from co-cultures, the co-culture was centrifuged at 3,000 rcf for 5 min at room temperature, to pellet host cells. Supernatant containing suspended Saccharibacteria was then collected and filtered twice using a pre-sterilized 5 μm mixed cellulose esters (MCE) filter. Filtrate was centrifuged at 15,000 rcf for 30 min at room temperature to pellet Saccharibacteria. The resulting pellet was resuspended in a small volume of TSYR supplemented with 10% (v/v) DMSO and stored at -80°C.

#### Saccharibacteria host compatibility testing

Purified populations of *Se* and *Nl* ML1 for host compatibility testing were generated by growing 100 mL co-cultures of each with their respective host strains according to the standard protocol described above. Co-cultures were passed through a 0.45 μm SFCA filter to remove host cells then spun at 80,000 rcf for 20 min to pellet Saccharibacteria. Supernatant was removed and cell pellets were resuspended in 1 mL fresh medium. Purified Saccharibacteria cells were then added to OD_600_ = 0.2 cultures of *Ai* and *Pp* (compatible host for *Nl*) at an MOI of 0.2. Cultures were then incubated statically at 37°C under ambient air enriched with 5% CO_2_ for up to 72 hr, and populations of *Se* and *Nl* were monitored over time using qPCR.

#### Whole genome sequencing

Genomic DNA of *Se*, *Nl* ML1and *Ai* F0345 was isolated using the Wizard HMW DNA Extraction kit (Promega). Sequencing was performed on Illumina iSeq and MiSeq and Oxford Nanopore (ONT) MinION instruments after standard sequencing library preparation protocols (Illumina and Oxford Nanopore). De-novo assemblies were generated using the Trycyler pipeline.^62^ Specifically, ONT long reads were filtered using Filtlong v0.2.1 (https://github.com/rrwick/Filtlong) with Illumina re–rence reads, using --keep_percent 95 to retain approximately 95% of the reads. Long reads were then subsampled into 12 bins and assembled into 12 assemblies using the Flye v2.9 (https://github.com/fenderglass/Flye/releases/tag/2.9. Accessed 4 October 2021), Raven v1.8.1^63^ Miniasm v0.3r179^64^ and Minipolish v0.1.3 (https://github.com/rrwick/Minipolish/blob/main/miniasm_and_minipolish.sh) assemblers. Assembly contigs were manually curated and then reconciled using Trycycler v0.5.2.^62^ A consensus assembly for each bacterium was generated and then polished with Illumina short reads using Polypolish v0.5.0^62^ and POLCA from MaSuRCA v4.0.9.^65^ Initial annotations were generated using PROKKA v1.14.5.^66^

To sequence clonal transformed *Se* and *Nl* TM7x (see Results and below), genomic DNA was isolated from frozen pellets of the purified Saccharibacteria by re-suspending in Buffer PB (Qiagen) to a total volume of 500 μL, adding 250 μL of 0.1mm zirconia/silica beads (BioSpec Catalog # 11079101z), 250 μL of 20% SDS, and 550 μL of phenol:chloroform:IAA (25:24:1) (Invitrogen Catalog #15593-031), and bead-beating in a Mini-BeadBeater-16 (Biospec Model 607) with settings 3450 RPM, 115V, 10A, and ½ HP, for four 30-second intervals, each followed by cooling on ice for 1 minute. Purification of the DNA was performed by applying the aqueous phase directly to a DNeasy Blood & Tissue Prep Kit (Qiagen) purification column and following the recommended protocol for washing and elution. Sequencing was performed on an Illumina iSeq using standard library preparation protocols (Illumina). Reads were mapped to the assembled *Se* ML1 genome or the *Nl* TM7x genome (GenBank: NZ_CP007496) using minimap2 and variants were called using LoFreq v2.

#### Phylogenetic analysis

The *Se* and *Nl* ML1 genomes were phylogenetically placed using whole genome information. A genome tree was generated from these newly isolated strain genomes with a manually curated set of high-quality Patescibacteria genomes (complete and partial) and metagenome assembled genomes (MAGS) to remove any contaminants deposited with the original assemblies as described previously.^16^ This species tree was constructed using a set of 50 core, universal proteins defined by COG (Clusters of Orthologous Groups) gene families with KBase.^72^ Sequences of each of the 50 selected proteins were individually aligned using MUSCLE, then concatenated into a curated single curated multiple sequence alignment (Data S2). The alignments were trimmed using BGME^67^ (default settings with gap rate cut-off 0.1) to remove poorly aligned sections resulting in 6042 columns, 3949 distinct patterns, 2686 parsimony-informative, 1525 singleton sites and 1831 constant sites (Data S3). A phylogenetic tree was reconstructed from this concatenated and trimmed alignment using IQtree (-st AA -m TEST -bb 1000 -alrt 1000).^68^ Genomes were annotated with the latest GTDB taxonomy (Release 214).^7^

#### Measuring hygromycin sensitivities of *Ai* and *Se*

Hygromycin sensitivity of *Ai* and *Se* was measured in liquid co-cultures. Duplicate co-cultures were initiated by mixing purified *Se* with *Ai* at an OD_600_ of 0.2 in TSY and at an approximate cellular ratio of 1:2 (*Se*:*Ai*). The co-cultures were divided into multiple aliquots in 96-well culture plates and hygromycin was added to the final concentrations shown in Figure S1. The plates were covered with Breath-Easy sealing membrane (Sigma Z380059) and incubated without lids at 37°C with 5% CO_2_. At 1 day and 3 days, the cells within individual wells were pelleted (>15 min at >15,000 rcf) and stored at -20°C for later genomic DNA isolation and qPCR-based quantification of *Se* and *Ai* (see below).

#### Design and generation of cassettes for heterologous gene expression in *Se* and *Nl*

Cassettes for heterologous gene expression in *Se* and *Nl* ML1were designed by appending promoter and terminator sequences from the *Nl* TM7x genome^13^ to the 5’ and 3’ ends, respectively, of ORFs codon optimized for *Se*. The promoter and terminator elements were sourced from *Nl* TM7x rather than *Se* to reduce the likelihood of off-target integration at corresponding *Se* loci. The *Nl* TM7x promoters were chosen from genes expected to be highly and constitutively expressed, *tuf* and *rpsJ*. *Se* codon usage was calculated using the Dynamic Codon Biaser (DCB).^73^ Heterologous ORFs were optimized to match relative codon usage frequencies found in *Se*, but omitting codons with less than 10% usage. Heterologous genes utilized included *hph* (hygromycin B phosphotransferase) from *Streptomyces hygroscopicus*, superfolder GFP (www.fpbase.org/protein/superfolder-gfp/), mCherry2 (www.fpbase.org/protein/mcherry2/) and NanoLuc Luciferase.^74^^,^^75^ The cassette used for transformation of *Nl* TM7x used the same codon-optimized *hph* ORF described above, but the promoter and terminator elements were sourced from the *Se tuf* gene. Table S4 reports the composition and complete sequences of the designed cassettes. Cassettes were obtained as gBlocks from Integrated DNA Technologies, Inc (IDT) or gene fragments from Twist Biosciences.

Linear fragments used for transformations were generated by adding *Se*, or *Nl* genomic sequences corresponding to the targeted insertion or allelic replacement sites to the left and right sides of a heterologous gene expression cassette or of two cassettes joined together. Overlap extension PCR was used to join gBlock cassettes and genomic fragments, the latter of which were individually amplified from *Se* or *Nl* genomic DNA. In some cases, complete fragments including genomic sequences were obtained as gBlocks from IDT or gene fragments from Twist Biosciences. Fragments used for flank-length tests were generated by amplification using larger fragments as templates followed by gel-purification. Table S4 reports the composition and complete sequences of the fragments utilized and primers used are listed in Table S3.

#### Genetic transformation of *Se* and *Nl* with targeted insertion and allelic replacement constructs

To prepare *Se*-*Ai* or *Nl* ML1*-Pp* co-cultures for transformation, 1-mL aliquots of a previously frozen co-culture (see methods on co-culturing) were thawed on ice and added to 9 mL of TSY supplemented with freshly cultured *Ai* to a final OD_600_ of 0.2, incubated statically for 2 d at 37°C in ambient air enriched with 5% CO_2_, then enlarged by the addition of 10 volumes of TSY and incubated anaerobically using the GasPak EZ Anaerobe Container System with Indicator (BD 260626 and 260001) at 37°C for 2 days with shaking at 160 rpm.

For transformation of *Se* or *Nl* ML1 with linear targeted insertion constructs, 0.22 - 0.3 mL aliquots of prepared co-culture were incubated statically with transforming DNA for 6 h at 37°C in ambient air enriched with 5% CO_2_ in culture tubes. The mixtures were subsequently enlarged by the addition of TSYR to approximately 5 mL and supplemented with recently passaged *Ai* (for *Se*) or *Pp* (for *Nl* ML1) to a final OD_600_ of approximately 0.06. This time point was designated “TF” (time zero after transformation). After removal of a sample for later qPCR analysis, the cultures were divided and one half was supplemented with hygromycin to 150 μg/mL. The cultures were then incubated as above for an additional 2-3 days. This time point was designated “P1” (end of initial passage). Cultures were subsequently serially passaged as many as four times by five-fold dilution into fresh TSYR supplemented with *Ai* (for *Se*) or *Pp* (for *Nl*) to a final OD_600_ of 0.06 and, for the hygromycin containing cultures, with additional hygromycin to 150 μg/mL. These passages (designated “P2”, “P3”, etc.) were each incubated for 2-3 days at 37°C in ambient air enriched with 5% CO_2_ and without agitation. Cultures were sampled at each passage by removing and pelleting (>15 min at >15,000 rcf) of 0.1 to 1 mL, and the pellets were stored at -20°C for later qPCR analysis.

For transformation of *Nl* TM7x with linear targeted insertion constructs, frozen *Nl* TM7x-*S*. *odontolytica* XH001 co-culture glycerol stock was inoculated into BHI media. Co-cultures were grown anaerobically and were passaged every 24 h until the end of passage 2 (P2). 0.9 mL co-cultures were incubated with 300 ng transforming DNA for 6 h at 37°C anaerobically. The rest of the transformation procedure is similar to that of *Se* and *Nl* ML1 described above, with the following differences: host supplemented during each passage was *S. odontolytica* XH001, media used was BHI, cultures were passaged every 24 h, and cultures were passaged to P4.

#### qPCR assays

We employed qPCR for quantification of Saccharibacteria and host populations in co-cultures, as well as to monitor *Se* transformation. For these assays, genomic DNA was isolated from frozen co-culture pellets using Instagene matrix (Bio-Rad) and quantitative PCR (qPCR) was performed using a CFX Connect Real-Time PCR Detection System (Bio-Rad). To quantify *Se* and *Ai*, amplification employed primers targeting *Se uvrB* or the 16S rRNA gene of *Ai* and was performed in 20 μL reactions with 1X SsoAdvanced Universal SYBR Green Supermix (Bio-Rad), 300 nM each primer and 4 μL template DNA (primer sequences provided in Table S3). Thermocycling conditions were 95°C for 5 min followed by 35-40 cycles of 95°C for 20 s, 60°C for 30 s and a read of fluorescence. To quantify transformed *Se* (insertions at NS1), amplification employed primer pairs targeting the insertion sequence and sequence adjacent to NS1 but outside of the linear transformation construct arms and was performed in 20 μL reactions with 1X Phusion HF Buffer, 0.2 mM dNTPs, 250 nM each primer, 0.5X SYBR Green I (ThermoFisher), 4 μL template DNA and 0.4 units of Phusion High-Fidelity DNA Polymerase (NEB) (primer sequences provided in Table S3). Thermocycling conditions were 98°C for 90 s followed by 35-40 cycles of 98°C for 15 s, 70°C for 20 s, 72°C for 90 s and a read of fluorescence. Melt curves were performed following each amplification to verify product homogeneity. Absolute target sequence abundance was determined by comparison to duplicate standard curve reactions performed in parallel with each assay. The standard curve templates were generated by serial dilution of previously amplified and gel-purified products quantified by Qubit (ThermoFisher).

#### Isolation of isogenic mutant co-cultures

To obtain co-cultures with isogenic mutant (transformed) *Se* and *Nl* TM7x, transformation mixtures grown for three passages (*Se*) or four passages (*Nl* TM7x) under hygromycin selection were serially diluted, plated on TSBY agar (*Se*) or BHI agar (*Nl* TM7x) without antibiotic and incubated for 5-7 days anaerobically. For *Se*, isolated colonies (representing *Ai* potentially colonized with *Se*) were picked into 0.2 mL TSYR supplemented with *Ai* at a final OD_600_ of 0.2 and incubated statically at 37°C in ambient air enriched with 5% CO_2_ for 2-4 days, then screened for the presence of *Se* and for the mutant (genomic insertion) and wild-type (no insertion) alleles by PCR (e.g., Figure 2A; primer sequences in Table S3). For *Nl* TM7x, isolated colonies (representing XH001 potentially colonized with *Nl* TM7x) were picked into 50 μL BHI media and 1 μL from which was used as template for PCR screening (Figures S2C and S2E). Cultures were incubated statically at 37°C anaerobically for 3 days with passaging every 24 h. Cultures containing pure mutant *Se* or *Nl* TM7x were frozen and/or further propagated for purification of the *Se* or *Nl* TM7x for WGS (see above).

#### Nanoluciferase assay

Nanoluciferase assays were performed using isogenic wild-type *Se-Ai* co-cultures and *Se* NS1::*nluc-hph2-Ai* co-cultures grown statically in ambient air supplemented with 5% CO_2_ at 37°C in TSY media for three passages as described above. To separate *Se* from host cells, 20 mL of each co-culture was passed through a 0.45 μM SCFM filter and the resulting filtrate was spun at 80,000 rcf for 30 min to pellet *Se*, supernatant was removed, and the pelleted *Se* was resuspended in 750 μL TSY. 100 μL of these purified *Se* cells or 100 μL of the corresponding *Se-Ai* co-cultures were mixed with 100 μL Nano-Glo Luciferase assay reagent (50:1 mixture of substrate:buffer, Promega N1110) in a 96-well plate. Luminescence signal indicative of nanoluciferase activity was detected using a Cytation 2 plate reader. Luminescence signal was later normalized by *Se* abundance in co-cultures and filtrates as measured by qPCR using *Se*-specific primers as described above.

#### Microscopy

Imaging was performed on a Nikon Eclipse Ti-E wide-field epi-fluorescence microscope, equipped with a sCMOS camera (Hamamatsu) and X-cite LED for fluorescence imaging. We imaged through a Nikon Plan Apo λ 60X 1.4 NA oil-immersion Ph3 objective. The microscope was controlled by NIS-Elements 3.30.02. Isogenic *Se* NS1::*mcherry-hph2-Ai* co-cultures and *Se* NS1::*sfgfp-hph2-Ai* co-cultures were grown statically in ambient air supplemented with 5% CO_2_ at 37°C in TSYR media as described above. Cell samples were spotted on a 3% (w/v) agarose pad made with TSYR media supplemented with 0.4% glucose placed on a microscope slide. The microscope chamber was heated to 37°C for time-lapse experiments. Time-lapse images were aligned and segmented with Omnipose using the phase contrast channel and the published bact_phase_omni model.^69^ Masks were manually linked and corrected for segmentation errors in regions of *Ai* cell overlap using Napari. Regions corresponding to *Se* were also removed to accurately track host *Ai* growth alone. For visualization purposes, fluorescence intensity was gamma-corrected to normalize background levels on a frame-by-frame basis while not distorting the *Se* signal. No bleaching correction was implemented. Figures 3A–3F and associated videos were generated using Python and annotated in Adobe Premiere.

#### Mutagenesis of Sac1a genomic DNA and transposon mutant library generation

The transposon used for *in vitro* transposition (here named T36) was generated by amplification of the *hph1* insertion cassette using the 5’-phosphorylated primers T36-ampF and T36-ampR, which add required 19-bp Tn*5* mosaic end sequences to each end of the amplicon, followed by purification using the Qiagen PCR Purification Kit with elution in TE (primer sequences provided in Table S3). *In vitro* transposition was performed in multiple 50-μl reactions, each containing 1.0 μg *Se* genomic DNA, 82 ng purified T36, 1X EZ-Tn5 Reaction Buffer and 3.4 U EZ-Tn*5* Transposase (Biosearch Technologies) with incubation for 2 hr at 37 °C. The reactions were stopped, DNA was ethanol precipitated, and gaps were repaired as described.^37^ Following gap repair, the DNA was purified using Qiagen PCR Purification Kit with elution in water after the columns were washed twice with Buffer PE.

Transformation of *Se* to generate transposon mutant pools was performed similarly to the procedure described above for transformation with targeted insertion fragments, but in quadruplicate, at larger scale and with modifications. Specifically, for each replicate the initial 6 hr incubation contained 3.3 μg of *in vitro* transposon-mutagenized genomic DNA and 0.75 mL of Ai-Se co-culture that had been concentrated 42-fold by centrifugation for 35 min at 15,000 rcf followed by re-suspension in a small volume of TSYR. Each replicate was then enlarged by adding 330 mL of TSYR supplemented with fresh *Ai* at a final OD_600_ of 0.12, then incubated for two additional hours before hygromycin addition. Concomitantly with hygromycin addition, MgCl_2_ (to 1 mM) and benzonase (Sigma) were added to 25 U/mL to degrade extracellular DNA, and the cultures were similarly supplemented with benzonase approximately every 24 h during passaging. After the initial 2-day growth passage (ending at time point T0), the culture was serially passaged three additional times by dilution of 160 mL culture into 640 mL in TSYR supplemented with *Ai* at OD_600_ of 0.15, hygromycin at 150 μg/mL and MgCl_2_ at 1 mM. These passages were each incubated statically for 2 days at 37°C in ambient air enriched with 5% CO_2_. Samples for Tn-seq analysis were taken at the end of the initial passage (170 mL per replicate, T0) and at the end passages 1, 2 and 3 (250 mL per replicate, T1-T3). These samples were treated with additional benzonase (50 U/mL) for 30 min, then EDTA was added to 15 mM and pellets were collected by centrifugation for 30 min at 15,000 rcf in multiple tubes per replicate and stored at -80°C.

#### Tn-seq library preparation and sequencing

Genomic DNA was isolated from frozen co-culture pellets using the bead-beating method described above (section on whole genome sequencing). Tn-seq libraries were prepared by the C-Tailing method as described.^76^ The transposon-specific primers utilized are listed in Table S3. Sequencing was performed in multiplex as 50-bp single-end reads on an Illumina MiniSeq with 25-40% PhiX spike-in.

Custom scripts (https://github.com/lg9/Tn-seq^37^^,^^76^) were used to process the Illumina reads. In brief, reads were first filtered for those displaying transposon end sequence as their initial bases (the sequencing primer was designed to anneal six bases from the end of the transposon). These reads were then mapped to the *Se* genome after removing the transposon end sequences. Reads per unique mapping position and orientation were tallied. For downstream analysis, the mapped read counts per site from all four replicates were combined for each time point, after normalizing the counts to 500,000 total reads per replicate. Read counts per gene were calculated by summing reads from all unique sites within a given gene.

#### *Se* gene essentiality analysis

Gene essentiality was determined at each time point (T0-T2) using the Hidden Markov Model method, HMM, part of the TRANSIT suite.^38^ Default parameters were used for the HMM analysis. HMM takes into account the read counts per insertion(s) in each gene to identify those with over- or under-represented insertion read counts. HMM classifies genes among four potential categories: essential, not essential, growth defect, or growth advantage. We focused on genes classified as essential. Although HMM was originally developed for analyzing Himar1 data, we were able to use HMM to analyze our Tn5 data by sequencing the *in vitro* transposon-mutagenized genomic DNA we originally used to transform *Se*. A total of 35,792 insertions were detected in that samples (with one or more reads). We expanded the universe of potential insertions by including insertions detected at any sampled time point (T0-T2). Thus, in contrast to the general idea of each nucleotide being a potential insertion site in Tn5 data (that would result in low saturation of the transposon mutant libraries and low performance of HMM),^77^ we estimated a total of 65,890 potential insertion sites in our Tn-seq experiments. The set of potential insertions was specified as part of the input for HMM runs (i.e.,.WIG file).

#### Transformations to validate gene essentiality predictions

To validate Tn-seq essentiality predictions, we designed constructs to replace target genes with *hph*. Each construct contained 335-bp sequences targeting the desired site flanking the *hph* expression cassette described above. 100 ng synthesize linear DNA or water were added to 300 μL wild-type *Se-Ai* co-cultures (4 biological replicates per condition) prepared according to our standard protocol, and transformation and subsequent passaging were performed using our general transformation protocol described above. At the end of passage 3, 1 mL of each transformed co-culture was treated with 1 mM MgCl_2_ and 25 U/mL benzonase for 30 min at 37°C to remove extracellular DNA. 15 mM EDTA was then added to stop the benzonase reaction, samples were centrifuged at 21,000 rcf for 1 hr and the resulting pellets were stored at -80°C. *Se* abundance was then measured by qPCR as described above.

#### Generation and transformation efficiency of *Se* Δ*comEC::hph*

A clonal population of *Se* Δ*comEC::hph* was generated by replacing *comEC* with our hygromycin resistance cassette following the transformation and clonal mutant isolation protocols described above. To assess *Se* Δ*comEC::hph* transformability, 50 ng of synthesized linear gene product for inserting unmarked *sfGFP* at NS1 (with 545 bp flanking regions) was used to transform 150 μL isogenic wild-type or Δ*comEC Se-Ai* co-cultures (4 biological replicates per condition). After 6.5 h of incubation between DNA and co-cultures at 37°C, the entire transformation culture was treated with 1 mM MgCl_2_ and 25 U/mL benzonase for 30 min at 37°C to degrade extracellular DNA. 15 mM EDTA was added to stop the benzonase reaction and the samples were centrifuged at 21,000 rcf for 1 hr and the resulting pellets were stored at -80°C. We then used qPCR to measure the abundance of transformed and total *Se* using NS1-sfGFP or *uvrB-* specific primers as described above.

#### Patescibacteria-enriched protein families analysis

To assess the protein family distribution of the *Se* and *Nl* genomes, we collected amino acid sequences for 22,977 protein clusters (protein families) from Meheust et al.^9^ These protein families were built through their occurrences across at least 5 distinct non-redundant or draft Patescibacteria, bacteria outside of Patescibacteria and Archaeal genomes. The presence or absence of any given family in *Se* or *Nl* were determined through all-vs-all protein sequence search of every protein in the genome against the database of protein family sequences. Protein sequence search was performed by using the MMseqs2 (version: 14-7e284) algorithm with the following parameters: module: easy-search; sensitivity (-s): 7.5; alignment coverage (-c): 0.5; greedy-best-hits: 1. The resulting presence or absence matrix was combined with the core family matrix of 921 protein families across 2890 genomes (collected from Meheust et al.^9^) for clustering analysis. A Jaccard distance based on the presence or absence of core families across *Se*, *Nl* and 2890 other genomes was calculated in R by using proxyC package (version: 0.3.3). Agglomerative hierarchical clustering was performed by using cluster package (version: 2.1.4) in R with the “wand” method. A hierarchical clustering heatmap was built using complexHeatmap package in R (version: 2.14.0) by plotting presence (black) or absence (white) of protein families (columns) in a given genome (rows). Enrichment or depletion of core protein family clusters in *Se* and *Nl* was performed using Fisher’s exact test in R with input table of presence or absence of protein families in the *Se* and *Nl* genomes. A threshold of Benjamini-Hochberg corrected p-values of < 5e-05 was used to determine the enrichment or depletion.

#### Sequence-based gene annotation of the *Se* genome

We used ProtNLM (https://github.com/google-research/google-research/tree/master/protnlm) to predict the functions of proteins in the Se proteome from sequence.^39^ A prediction of score ≥0.5 corresponds to >70% accuracy for proteins without close UniRef50 matches and ∼95% accuracy for proteins with close matches. We thus considered the 337 proteins with score ≥0.5 to have confident annotations (Table S2). Additionally, we mapped each protein to Pfam domains (Nov, 2021 release v35.0^46^) using hmmscan.^40^ We filtered the hit Pfam domains by full sequence E-value ≤ 1e-3 and domain-based CE-value ≤ 1e-5 or CI-value ≤ 1e-5, domain coverage ≥ 50% (coverage of aligned portion of domain to full length domain) and selected the top hit for each aligned region. Finally, we used KofamKOALA^70^ (version 2023-04-01) to conduct an HMM search against the KEGG Orthology (KO) database^78^ (release 106.0). We assigned broad functional categories based on KO to the 353 proteins which mapped to KOs above the adaptive significance threshold (E-value ≤ 0.001) which are computed for each KO family as described in Aramaki et al.^70^

#### Generation of multiple sequence alignments

To construct MSAs for each protein in the *Se* proteome, we initially used HHblits^44^ to search against UniRef (2022 release^46^) and BFD (2019 release^45^). For those MSAs that contained <500 sequences after this approach, we conducted extensive homology searches against metagenome databases similar to Anishchenko et al.^79^ We first converted the initial HHblits (E-value ≤ 1e-3) MSAs for each protein into a hidden Markov model (HMM) which we used as a seed sequence profile to search against metagenomic and metatranscriptomic sequences from JGI,^80^ MGnify,^81^ and UniRef with hmmsearch,^40^ respectively. Hits from JGI, MGnify, and UniRef were gathered and aligned to the query protein by phmmer and jackhmmer,^40^ to retain sequences with E-value ≤ 1e-5 at each stage. We removed columns containing gaps in the query sequence from the MSAs, which were then subjected to redundancy filtering at 95% sequence identity and 50% sequence coverage. We selected the deepest MSA for each protein (from HHblits against UniRef+BFD or phmmer/jackhmmer against metagenome) for downstream analysis.

#### Structure-based annotations

Using MSAs described above, we computed AlphaFold (AF) models (model_1 without structural template search, 10 recycles, and version 1.0 weights) for 852 of the 855 proteins (3 were excluded because of MSA depth or protein size).^43^ We then applied FoldSeek^47^ (v6.0, sensitivity 9.5, and alignment type 1) against AFdatabase50 (AF Protein Structure Database v4 clustered at 50% sequence identity and 80% coverage, accessed May 2023). We report the top 3 hits in Table S2 based on normalized TM-alignment score ≥ 0.5 using alignment type 1 (TM-score; which correlates well to fold-family level similarity^52^), and query coverage and target coverage ≥ 50%. To classify proteins into evolutionary contexts based on structure and sequence similarity, we used Domain Parser for AlphaFold Models (DPAM),^49^ and the full results are available at https://conglab.swmed.edu/ECOD_Se/Se_ECOD.html.

### Quantification and statistical analysis

Statistical significance in Figures 2E and 4D was assessed by unpaired two-tailed student’s t-tests between relevant samples. Statistical significance in Figure 4B was assessed by one-way ANOVA with Dunnett’s multiple comparison test compared to a no DNA control. Statistical details for each experiment can be found in the figure legends.

## Data Availability

• The complete genome sequences of *Se* ML1, *Nl* ML1and *Ai* F0345 have been deposited in GenBank under BioProject PRJNA957798 with accession numbers SAMN34266291, SAMN34266292 and SAMN34266293, respectively. Tn-seq data generated in this study have been deposited in the Sequence Reads Archive (SRA, BioProject PRJNA957798). These datasets are publicly available as of the date of publication. • This paper does not report original code. • Any additional information required to reanalyze the data reported in this paper is available from the lead contact upon request.

## Supplemental information

Table S1. Tn-seq data and analysis, related to Figure 4 Light gray shaded rows indicate class I essential genes, and dark gray shading indicates class II essential genes. Blue text indicates genes discussed in the text.

Table S2. Sequence and structure-based annotation of the *Se* genome, related to Figure 5

Table S3. Oligonucleotide primers employed in this study, related to STAR Methods and key resources table

Table S4. Heterologous gene expression cassettes and linear fragments used in Saccharibacteria transformations, related to STAR Methods and key resources table

## References

[1] Hug L.A., Baker B.J., Anantharaman K., Brown C.T., Probst A.J., Castelle C.J., Butterfield C.N., Hernsdorf A.W., Amano Y., Ise K. (2016). A new view of the tree of life. Nat. Microbiol..

[2] Marcy Y., Ouverney C., Bik E.M., Lösekann T., Ivanova N., Martin H.G., Szeto E., Platt D., Hugenholtz P., Relman D.A., Quake S.R. (2007). Dissecting biological "dark matter" with single-cell genetic analysis of rare and uncultivated TM7 microbes from the human mouth. Proc. Natl. Acad. Sci. USA.

[3] Katz M., Hover B.M., Brady S.F. (2016). Culture-independent discovery of natural products from soil metagenomes. J. Ind. Microbiol. Biotechnol..

[4] Rinke C., Schwientek P., Sczyrba A., Ivanova N.N., Anderson I.J., Cheng J.F., Darling A., Malfatti S., Swan B.K., Gies E.A. (2013). Insights into the phylogeny and coding potential of microbial dark matter. Nature.

[5] Coleman G.A., Davín A.A., Mahendrarajah T.A., Szánthó L.L., Spang A., Hugenholtz P., Szöllősi G.J., Williams T.A. (2021). A rooted phylogeny resolves early bacterial evolution. Science.

[6] Megrian D., Taib N., Jaffe A.L., Banfield J.F., Gribaldo S. (2022). Ancient origin and constrained evolution of the division and cell wall gene cluster in Bacteria. Nat. Microbiol..

[7] Parks D.H., Chuvochina M., Waite D.W., Rinke C., Skarshewski A., Chaumeil P.A., Hugenholtz P. (2018). A standardized bacterial taxonomy based on genome phylogeny substantially revises the tree of life. Nat. Biotechnol..

[8] Brown C.T., Hug L.A., Thomas B.C., Sharon I., Castelle C.J., Singh A., Wilkins M.J., Wrighton K.C., Williams K.H., Banfield J.F. (2015). Unusual biology across a group comprising more than 15% of domain Bacteria. Nature.

[9] Méheust R., Burstein D., Castelle C.J., Banfield J.F. (2019). The distinction of CPR bacteria from other bacteria based on protein family content. Nat. Commun..

[10] Castelle C.J., Brown C.T., Anantharaman K., Probst A.J., Huang R.H., Banfield J.F. (2018). Biosynthetic capacity, metabolic variety and unusual biology in the CPR and DPANN radiations. Nat. Rev. Microbiol..

[11] Ji Y., Zhang P., Zhou S., Gao P., Wang B., Jiang J. (2022). Widespread but poorly understood bacteria: candidate phyla radiation. Microorganisms.

[12] Batinovic S., Rose J.J.A., Ratcliffe J., Seviour R.J., Petrovski S. (2021). Cocultivation of an ultrasmall environmental parasitic bacterium with lytic ability against bacteria associated with wastewater foams. Nat. Microbiol..

[13] He X., McLean J.S., Edlund A., Yooseph S., Hall A.P., Liu S.Y., Dorrestein P.C., Esquenazi E., Hunter R.C., Cheng G. (2015). Cultivation of a human-associated TM7 phylotype reveals a reduced genome and epibiotic parasitic lifestyle. Proc. Natl. Acad. Sci. USA.

[14] Kuroda K., Yamamoto K., Nakai R., Hirakata Y., Kubota K., Nobu M.K., Narihiro T. (2022). Symbiosis between Candidatus Patescibacteria and Archaea discovered in wastewater-treating bioreactors. mBio.

[15] Yakimov M.M., Merkel A.Y., Gaisin V.A., Pilhofer M., Messina E., Hallsworth J.E., Klyukina A.A., Tikhonova E.N., Gorlenko V.M. (2022). Cultivation of a vampire: 'Candidatus Absconditicoccus praedator'. Environ. Microbiol..

[16] McLean J.S., Bor B., Kerns K.A., Liu Q., To T.T., Solden L., Hendrickson E.L., Wrighton K., Shi W., He X. (2020). Acquisition and adaptation of ultra-small parasitic reduced genome bacteria to mammalian Hosts. Cell Rep..

[17] Bor B., Bedree J.K., Shi W., McLean J.S., He X. (2019). Saccharibacteria (TM7) in the human oral microbiome. J. Dent. Res..

[18] Brinig M.M., Lepp P.W., Ouverney C.C., Armitage G.C., Relman D.A. (2003). Prevalence of bacteria of division TM7 in human subgingival plaque and their association with disease. Appl. Environ. Microbiol..

[19] Ouverney C.C., Armitage G.C., Relman D.A. (2003). Single-cell enumeration of an uncultivated TM7 subgroup in the human subgingival crevice. Appl. Environ. Microbiol..

[20] Paster B.J., Boches S.K., Galvin J.L., Ericson R.E., Lau C.N., Levanos V.A., Sahasrabudhe A., Dewhirst F.E. (2001). Bacterial diversity in human subgingival plaque. J. Bacteriol..

[21] Chipashvili O., Utter D.R., Bedree J.K., Ma Y., Schulte F., Mascarin G., Alayyoubi Y., Chouhan D., Hardt M., Bidlack F. (2021). Episymbiotic Saccharibacteria suppresses gingival inflammation and bone loss in mice through host bacterial modulation. Cell Host Microbe.

[22] Adler C.J., Dobney K., Weyrich L.S., Kaidonis J., Walker A.W., Haak W., Bradshaw C.J., Townsend G., Sołtysiak A., Alt K.W. (2013). Sequencing ancient calcified dental plaque shows changes in oral microbiota with dietary shifts of the Neolithic and Industrial revolutions. Nat. Genet..

[23] Bor B., McLean J.S., Foster K.R., Cen L., To T.T., Serrato-Guillen A., Dewhirst F.E., Shi W., He X. (2018). Rapid evolution of decreased host susceptibility drives a stable relationship between ultrasmall parasite TM7x and its bacterial host. Proc. Natl. Acad. Sci. USA.

[24] Nie J., Utter D.R., Kerns K.A., Lamont E.I., Hendrickson E.L., Liu J., Wu T., He X., McLean J., Bor B. (2022). Strain-level variation and diverse Host Bacterial Responses in Episymbiotic Saccharibacteria. mSystems.

[25] Xie B., Wang J., Nie Y., Tian J., Wang Z., Chen D., Hu B., Wu X.L., Du W. (2022). Type IV pili trigger episymbiotic association of Saccharibacteria with its bacterial host. Proc. Natl. Acad. Sci. USA.

[26] Bor B., Collins A.J., Murugkar P.P., Balasubramanian S., To T.T., Hendrickson E.L., Bedree J.K., Bidlack F.B., Johnston C.D., Shi W. (2020). Insights obtained by culturing saccharibacteria with their bacterial Hosts. J. Dent. Res..

[27] Cross K.L., Campbell J.H., Balachandran M., Campbell A.G., Cooper C.J., Griffen A., Heaton M., Joshi S., Klingeman D., Leys E. (2019). Targeted isolation and cultivation of uncultivated bacteria by reverse genomics. Nat. Biotechnol..

[28] Albertsen M., Hugenholtz P., Skarshewski A., Nielsen K.L., Tyson G.W., Nielsen P.H. (2013). Genome sequences of rare, uncultured bacteria obtained by differential coverage binning of multiple metagenomes. Nat. Biotechnol..

[29] Podar M., Abulencia C.B., Walcher M., Hutchison D., Zengler K., Garcia J.A., Holland T., Cotton D., Hauser L., Keller M. (2007). Targeted access to the genomes of low-abundance organisms in complex microbial communities. Appl. Environ. Microbiol..

[30] He X. (2023). Culture-based approaches to studying "microbial dark matter". Proc. Natl. Acad. Sci. USA.

[31] Stackebrandt E., Goebel B.M. (1994). Taxonomic note: A place for DNA-DNA reassociation and 16S rRNA sequence analysis in the present species definition in bacteriology. Int. J. Syst. Evol. Microbiol..

[32] Damke P.P., Celma L., Kondekar S.M., Di Guilmi A.M., Marsin S., Dépagne J., Veaute X., Legrand P., Walbott H., Vercruyssen J. (2022). ComFC mediates transport and handling of single-stranded DNA during natural transformation. Nat. Commun..

[33] Dubnau D., Blokesch M. (2019). Mechanisms of DNA uptake by naturally competent bacteria. Annu. Rev. Genet..

[34] Sharma D.K., Misra H.S., Bihani S.C., Rajpurohit Y.S. (2023). Biochemical properties and roles of DprA protein in bacterial natural transformation, virulence, and Pilin variation. J. Bacteriol..

[35] Johnston C., Martin B., Fichant G., Polard P., Claverys J.P. (2014). Bacterial transformation: distribution, shared mechanisms and divergent control. Nat. Rev. Microbiol..

[36] Tian J., Utter D.R., Cen L., Dong P.T., Shi W., Bor B., Qin M., McLean J.S., He X. (2022). Acquisition of the arginine deiminase system benefits epiparasitic Saccharibacteria and their host bacteria in a mammalian niche environment. Proc. Natl. Acad. Sci. USA.

[37] Gallagher L.A., Bailey J., Manoil C. (2020). Ranking essential bacterial processes by speed of mutant death. Proc. Natl. Acad. Sci. USA.

[38] DeJesus M.A., Ambadipudi C., Baker R., Sassetti C., Ioerger T.R. (2015). Transit--A software tool for Himar1 TnSeq analysis. PLOS Comp. Biol..

[39] Gane A., Bileshi M.L., Dohan D., Speretta E., Heliou A., Meng-Papaxanthos L., Zellner H., Brevdo E., Parikh A., Martin M.J. (2022). ProtNLM: model-based natural language protein annotation. Preprint.

[40] Eddy S.R. (2011). Accelerated profile HMM Searches. PLoS Comp. Biol..

[41] Mistry J., Chuguransky S., Williams L., Qureshi M., Salazar G.A., Sonnhammer E.L.L., Tosatto S.C.E., Paladin L., Raj S., Richardson L.J. (2021). Pfam: the protein families database in 2021. Nucleic Acids Res..

[42] Baek M., DiMaio F., Anishchenko I., Dauparas J., Ovchinnikov S., Lee G.R., Wang J., Cong Q., Kinch L.N., Schaeffer R.D. (2021). Accurate prediction of protein structures and interactions using a three-track neural network. Science.

[43] Jumper J., Evans R., Pritzel A., Green T., Figurnov M., Ronneberger O., Tunyasuvunakool K., Bates R., Žídek A., Potapenko A. (2021). Highly accurate protein structure prediction with AlphaFold. Nature.

[44] Remmert M., Biegert A., Hauser A., Söding J. (2011). HHblits: lightning-fast iterative protein sequence searching by HMM-HMM alignment. Nat. Methods.

[45] Steinegger M., Mirdita M., Söding J. (2019). Protein-level assembly increases protein sequence recovery from metagenomic samples manyfold. Nat. Methods.

[46] Suzek B.E., Huang H., McGarvey P., Mazumder R., Wu C.H. (2007). UniRef: comprehensive and non-redundant UniProt reference clusters. Bioinformatics.

[47] van Kempen M., Kim S.S., Tumescheit C., Mirdita M., Lee J., Gilchrist C.L.M., Söding J., Steinegger M. (2023). Fast and accurate protein structure search with Foldseek. Nat. Biotechnol..

[48] Cheng H., Schaeffer R.D., Liao Y., Kinch L.N., Pei J., Shi S., Kim B.H., Grishin N.V. (2014). ECOD: an evolutionary classification of protein domains. PLoS Comp. Biol..

[49] Zhang J., Schaeffer R.D., Durham J., Cong Q., Grishin N.V. (2023). DPAM: A domain parser for AlphaFold models. Protein Sci..

[50] Ellison C.K., Dalia T.N., Vidal Ceballos A., Wang J.C., Biais N., Brun Y.V., Dalia A.B. (2018). Retraction of DNA-bound type IV competence pili initiates DNA uptake during natural transformation in Vibrio cholerae. Nat. Microbiol..

[51] Hepp C., Maier B. (2016). Kinetics of DNA uptake during transformation provide evidence for a translocation ratchet mechanism. Proc. Natl. Acad. Sci. USA.

[52] Moreira D., Zivanovic Y., López-Archilla A.I., Iniesto M., López-García P. (2021). Reductive evolution and unique predatory mode in the CPR bacterium Vampirococcus lugosii. Nat. Commun..

[53] Murugkar P.P., Collins A.J., Chen T., Dewhirst F.E. (2020). Isolation and cultivation of candidate phyla radiation Saccharibacteria (TM7) bacteria in coculture with bacterial hosts. J. Oral Microbiol..

[54] Cressler C.E., McLEOD D.V., Rozins C., VAN DEN Hoogen J., Day T. (2016). The adaptive evolution of virulence: a review of theoretical predictions and empirical tests. Parasitology.

[55] Egido J.E., Costa A.R., Aparicio-Maldonado C., Haas P.J., Brouns S.J.J. (2022). Mechanisms and clinical importance of bacteriophage resistance. FEMS Microbiol. Rev..

[56] Zúñiga M., Pérez G., González-Candelas F. (2002). Evolution of arginine deiminase (ADI) pathway genes. Mol. Phylogenet. Evol..

[57] Trchounian A., Trchounian K. (2019). Fermentation revisited: how do microorganisms survive under energy-limited conditions?. Trends Biochem. Sci..

[58] Sekiya M. (2022). Proton pumping ATPases: rotational catalysis, physiological roles in oral pathogenic bacteria, and inhibitors. Biol. Pharm. Bull..

[59] Dewhirst F.E., Chen T., Izard J., Paster B.J., Tanner A.C., Yu W.H., Lakshmanan A., Wade W.G. (2010). The human oral microbiome. J. Bacteriol..

[60] Batty I. (1958). Actinomyces odontolyticus, a new species of actinomycete regularly isolated from deep carious dentine. J. Pathol. Bacteriol..

[61] Nikolaitchouk N., Hoyles L., Falsen E., Grainger J.M., Collins M.D. (2000). Characterization of Actinomyces isolates from samples from the human urogenital tract: description of Actinomyces urogenitalis sp. nov.. Int. J. Syst. Evol. Microbiol..

[62] Wick R.R., Judd L.M., Cerdeira L.T., Hawkey J., Méric G., Vezina B., Wyres K.L., Holt K.E. (2021). Trycycler: consensus long-read assemblies for bacterial genomes. Genome Biol..

[63] Vaser R., Šikić M. (2021). Time- and memory-efficient genome assembly with Raven. Nat Comput. Sci..

[64] Li H. (2016). Minimap and miniasm: fast mapping and de novo assembly for noisy long sequences. Bioinformatics.

[65] Zimin A.V., Puiu D., Luo M.C., Zhu T., Koren S., Marçais G., Yorke J.A., Dvořák J., Salzberg S.L. (2017). Hybrid assembly of the large and highly repetitive genome of Aegilops tauschii, a progenitor of bread wheat, with the MaSuRCA mega-reads algorithm. Genome Res..

[66] Seemann T. (2014). Prokka: rapid prokaryotic genome annotation. Bioinformatics.

[67] Criscuolo A., Gribaldo S. (2010). BMGE (Block Mapping and Gathering with Entropy): a new software for selection of phylogenetic informative regions from multiple sequence alignments. BMC Evol. Biol..

[68] Trifinopoulos J., Nguyen L.T., von Haeseler A., Minh B.Q. (2016). W-IQ-TREE: a fast online phylogenetic tool for maximum likelihood analysis. Nucleic Acids Res..

[69] Cutler K.J., Stringer C., Lo T.W., Rappez L., Stroustrup N., Brook Peterson S., Wiggins P.A., Mougous J.D. (2022). Omnipose: a high-precision morphology-independent solution for bacterial cell segmentation. Nat. Methods.

[70] Aramaki T., Blanc-Mathieu R., Endo H., Ohkubo K., Kanehisa M., Goto S., Ogata H. (2020). KofamKOALA: KEGG ortholog assignment based on profile HMM and adaptive score threshold. Bioinformatics.

[71] Collins A.J., Murugkar P.P., Dewhirst F.E. (2021). Establishing stable binary cultures of symbiotic saccharibacteria from the oral cavity. J. Vis. Exp..

[72] Arkin A.P., Cottingham R.W., Henry C.S., Harris N.L., Stevens R.L., Maslov S., Dehal P., Ware D., Perez F., Canon S. (2018). KBase: the United States Department of Energy systems biology KnowledgeBase. Nat. Biotechnol..

[73] Dehlinger B., Jurss J., Lychuk K., Putonti C. (2021). The Dynamic Codon Biaser: calculating prokaryotic codon usage biases. Microb. Genom..

[74] Hall M.P., Unch J., Binkowski B.F., Valley M.P., Butler B.L., Wood M.G., Otto P., Zimmerman K., Vidugiris G., Machleidt T. (2012). Engineered luciferase reporter from a deep sea shrimp utilizing a novel imidazopyrazinone substrate. ACS Chem. Biol..

[75] Zalacain M., González A., Guerrero M.C., Mattaliano R.J., Malpartida F., Jiménez A. (1986). Nucleotide sequence of the hygromycin B phosphotransferase gene from Streptomyces hygroscopicus. Nucleic Acids Res..

[76] Gallagher L.A. (2019). Methods for TN-seq analysis in Acinetobacter baumannii. Methods Mol. Biol..

[77] Ioerger T.R. (2022). Analysis of gene essentiality from TnSeq data using transit. Methods Mol. Biol..

[78] Kanehisa M., Goto S. (2000). KEGG: kyoto encyclopedia of genes and genomes. Nucleic Acids Res..

[79] Anishchenko I., Baek M., Park H., Hiranuma N., Kim D.E., Dauparas J., Mansoor S., Humphreys I.R., Baker D. (2021). Protein tertiary structure prediction and refinement using deep learning and Rosetta in CASP14. Proteins.

[80] Chen I.A., Chu K., Palaniappan K., Ratner A., Huang J., Huntemann M., Hajek P., Ritter S., Varghese N., Seshadri R. (2021). The IMG/M data management and analysis system v.6.0: new tools and advanced capabilities. Nucleic Acids Res..

[81] Mitchell A.L., Almeida A., Beracochea M., Boland M., Burgin J., Cochrane G., Crusoe M.R., Kale V., Potter S.C., Richardson L.J. (2020). MGnify: the microbiome analysis resource in 2020. Nucleic Acids Res..

